# Design, synthesis and biological evaluation of a new thieno[2,3-*d*]pyrimidine-based urea derivative with potential antitumor activity against tamoxifen sensitive and resistant breast cancer cell lines

**DOI:** 10.1080/14756366.2020.1804383

**Published:** 2020-08-11

**Authors:** Marwa Sharaky, Marwa Kamel, Marwa A. Aziz, Mervat Omran, Monira M. Rageh, Khaled A. M. Abouzid, Samia A. Shouman

**Affiliations:** a Department of Cancer Biology, Pharmacology Unit, National Cancer Institute, Cairo University, Cairo, Egypt; b Department of Pharmaceutical Chemistry, Faculty of Pharmacy, Ain Shams University, Abbassia, Cairo, Egypt; c Department of Biophysics, Faculty of Science, Cairo University, Giza, Egypt; d Department of Organic and Medicinal Chemistry, Faculty of Pharmacy, University of Sadat City, Menoufia, Egypt

**Keywords:** Thieno[2,3-d]pyrimidine-based urea derivative, breast cancer, VEGFR-2 kinase, apoptosis makers, antiangiogenesis, anti-inflammatory

## Abstract

Breast cancer (BC) and endocrine resistance to chemotherapy are challenging problems where angiogenesis plays fundamental roles. Thus, targeting of VEGFR-2 signalling pathway has been an attractive approach. In this study, we synthesised a new sorafenib analogue, thieno[2,3-*d*]pyrimidine based urea derivative, **KM6**. It showed 65% inhibition of VEGF2 tyrosine kinase activity and demonstrated a potential antitumor activity in TAM-resistant, LCC2, and its parental MCF7 BC cells. **KM6** retained the sensitivity of LCC2 through upregulation of key enzymes of apoptosis and proteins of cell death including caspases 3, 8, 9, P53, BAX/BCL-2 ratio and LDH in media. It downregulated mRNA expression of Ki-67, survivin, Akt, and reduced levels of ROS and glucose uptake. Moreover, **KM6** reduced the levels of inflammation markers PGE2, COX2, IL-1β and IL6 and metastasis markers MMP-2 and MMP-9. In conclusion, **KM6** is a promising compound for ER + and TAM-resistant BC with many potential antitumor and polypharmacological mechanisms.

## Graphical Abstract

**Figure:**
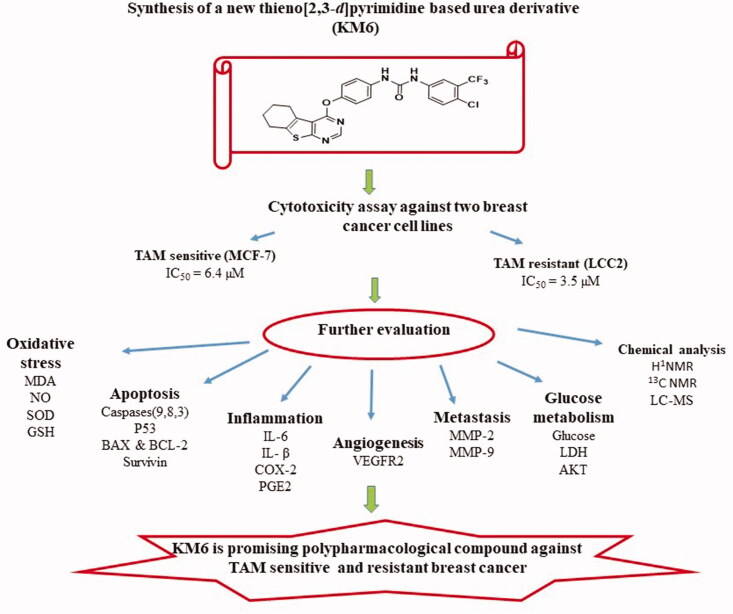

## Introduction

The World Health Organisation considers breast cancer (BC) as the most common malignant tumour among women^1^. It is expected that the number of new cases will increase from 14 million in 2012 to 22 million by 2022^2^. BC involves a complex microenvironment with unbalanced biological characteristics^3^. The majority of malignant breast tumours are classified as oestrogen receptor positive (ER+) BC where antiestrogens play a key role in the endocrine therapy of this type. The nonsteroidal compound Tamoxifen (TAM) is the most extensively used antiestrogen. However, acquired resistance to TAM is a serious therapeutic problem among ER + BC patients^4^. TAM resistance arises because of irregular signalling pathways within the tumour microenvironment and it has been illustrated that angiogenesis plays a fundamental role in this resistance^5^.

Angiogenesis ensures sufficient supply of oxygen and nutrients to proliferating cells, resulting in tumour progression and metastasis^6^. Tumour angiogenesis is regulated by a finely tuned equilibrium between proangiogenic and antiangiogenic factors, produced by host and tumour cells, including vascular endothelial growth factor (VEGF)^7^. VEGF family of tyrosine kinase receptors includes three protein receptors: VEGFR-1 (FLT1), VEGFR-2 (KDR/FLK1) and VEGFR-3 (FLT4). VEGFR-1 and VEGFR-2 generally mediate angiogenesis while VEGFR-3 regulates lymphangiogenesis. Specifically, suppression of VEGF/VEGFR signalling pathway is a remarkable therapeutic target for inhibiting tumour angiogenesis and subsequent tumour growth. VEGFR-2 is the most important transducer of VEGF-dependent angiogenesis, and therefore, treatment with VEGFR-2 inhibitors may be a way to target tumour growth^8^^,^^9^ including BC^10^. In addition, it has been suggested that utilising VEGFR inhibitors is a way to overcome endocrine resistance at multiple levels^11^. For example, ruxolitinib has been proposed as a new therapeutic targeting agent for TAM-resistant BC through inhibiting VEGF mRNA expression and transcriptional activity^12^. Sorafenib is a diaryl urea derivative that acts as a multityrosine kinase inhibitor. It inhibits VEGFR-2, PDGFR, RAF, FLIT-3 and C-KIT and is used in treatment of hepatocellular and renal cell carcinomas^13^. It has been investigated in the treatment of TAM-resistant BC and showed remarkable sensitisation to TAM^14^. By modification of sorafenib structure, several analogues have been synthesised to improve its cytotoxic and antiangiogenic activities. These modifications include replacement of urea with thiourea^15^, ether link with thioether^16^, pyridyl carboxamide moiety with other scaffolds as pyrazolo pyrimidine I and quinazoline II^17^^,^^18^. Some of these analogues exhibited better activity than sorafenib but none of them has been examined against TAM resistance. Thieno [2,3-*d*]pyrimidine ring system constitutes an attractive chemical scaffold with several reported biological activities e.g. inhibition of inflammatory-related diseases, acting as potent ligands for 5-HT3 and/or 5-HT4 receptors and others^19–22^. Moreover, it is considered a bioisostere of 4-aminoquinazoline core that comprises the structures of potent anticancer drugs as gefitinib and erlotinib. Several literatures reported that thieno[2,3-*d*]pyrimidines fused with lipophilic cycloalkyl ring (III, IV) potentiate the anticancer activity^23–25^. Consequently, the present investigation is concerned with the synthesis of a new sorafenib analogue **KM6** with benzothienopyrmidine ring system replacing the pyridyl carboxamide moiety in sorafenib. The new derivative **KM6** was tested for its cytotoxic activity against different BC cell lines namely MCF7, T47D, MDA-MB and LCC2 (TAM-resistant cells). Additional experiments were also performed to explore the different mechanisms where **KM6** exerts its cytotoxic activity. 
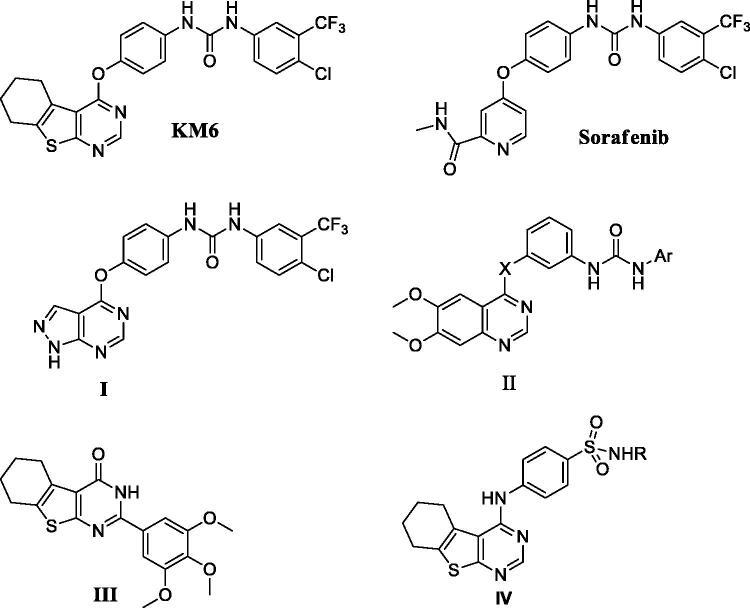


## Materials and methods

All starting materials, reagents and solvents were purchased from (Sigma Aldrich Chemical Co., St. Louis) and used without further purification. Monitoring of chemical reactions was performed by analytical thin layer chromatography (TLC), on silica gel 60 F254 packed on Aluminium sheets, purchased from (Merck, New Jersey) and were visualised using U.V. lamp (254 nm). Column chromatography was conducted on silica 60 (35–70 microns) eluent gradient hexane: ethyl acetate 90:10 to 85:15). Melting points were determined by (Stuart Scientific apparatus, UK). ^1^H-NMR spectra were recorded on a Bruker 400 MHz spectrometer in δ scale (ppm) using DMSO as a solvent and TMS as an internal standard. ^13^C NMR spectra was run at Joel 100 MHz spectrometer in δ scale *J*(Hz) using DMSO as a solvent and TMS as the internal standard. The LC–MS-MS system consisted of a Agilent 1200 HPLC system (Agilent technologies, CA) with a quaternary gradient pump (Agilent 1260 infinity), an online vacuum degasser, a column oven and an autosampler (Agilent 1260 infinity), coupled to a ABSCIEX Q TRAP 3200 mass spectrometer (ABSCIEX, Germany) equipped with an electrospray ionisation (ESI) interface. Data acquisition was performed with analyst 4.0 software (ABSCIEX). Separation was performed using a Waters XBridge-C18-5 µm (2.1 × 150 mm Column, Agilent, Germany) reversed phase analytical column (Agilent, CA). The mobile phase was pumped at flow rate of 300 µl/min and it consisted of 0.1% formic acid/10 mM ammonium format (solvent A) and 100% acetonitrile (solvent B). Over all run time was 12 min. The mass spectrometer was operated in the positive ESI mode with spray voltage set at 4 kV, at a temperature of 350 °C and a sweep gas flow of 20 L/h. Calculations were completed by the Multiquant software programme. Serial dilutions of standards were prepared at concentrations ranging from 62.5 to 1000 ng/ml for sorafinib and **KM6** to make calibration curves^26^^,^^27^.

### Drugs and compounds

***TAM citrate*** was obtained from (Amria Pharmaceuticals Company, Alexandria, Egypt). It is a white powder stored at room temperature. TAM was dissolved in dimethylsulfoide (DMSO) and prepared as stock solution at concentration 10 mM and was kept at −20 °C. Immediately before use, it was serially diluted in RPMI1640 to yield a concentration range of 2–80 µM.

#### Synthesis and characterisation of KM6

**Scheme 1.**
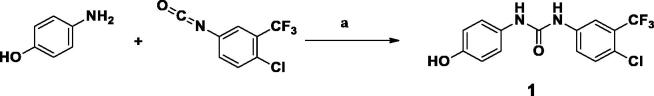
Preparation of diaryl urea derivative: Reagents and conditions: (a) 1,4-dioxane, room temperature, 1 h, 75%.

**Scheme 2.**
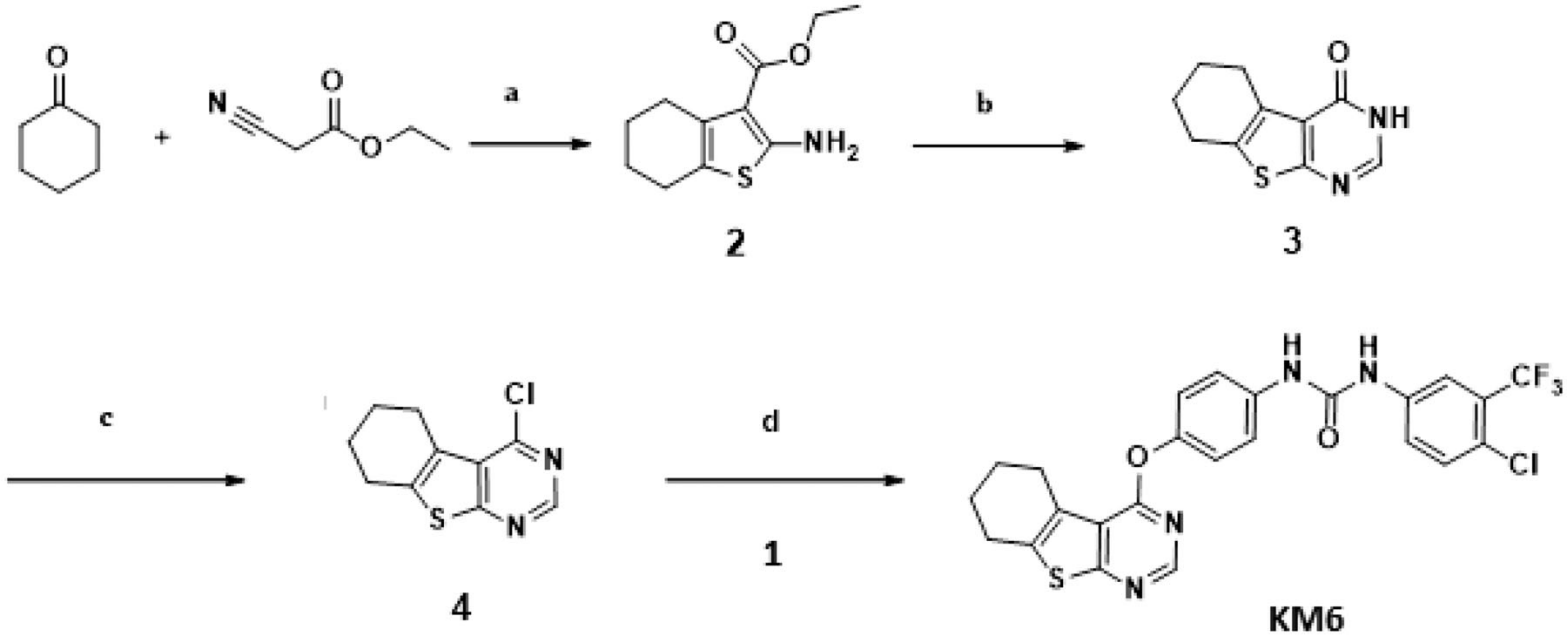
Preparation of **KM6**: Reagents and conditions: (a) S, piperidine, water bath at 50–60 °C, 16 h,70% (b) HCONH_2_, reflux, 3 h, 81% (c) POCl_3_, reflux, 3 h, 76% (d) Acetonitrile, urea derivative (1), Cs_2_CO_3_, 60 °C, 6 h, 66%.

As described in Schemes 1 and 2, synthesis of **KM6** was accomplished by a convergent synthesis through condensation of the diaryl urea derivative **(1)** with 4-chloro-5,6,7,8-tetrahydrobenzo[4–5]thieno[2,3-*d*]pyrimidine derivative **(4)**. The diaryl urea **(1)** was obtained by reaction of p-aminophenol with 4-chloro-3(trifluoromethyl) phenyl isocyanate in dry 1,4-dioxane^28^ (Scheme 1). Derivative **(4)** was synthesised via three reaction steps starting by reaction of cyclohexanone, ethyl cyanoacetate and elemental sulfur in the presence of piperidine (Gewald reaction) to give the 2-amino tetrahydrobenzothiophene derivative **(2)**. Compound **(2)** upon cyclisation with formamide gave the tetrahydrobenzothienopyrimidine derivative **(3)** which was chlorinated to afford compound (**4)**^29–31^ (Scheme 2). ^1^H-NMR of **KM6** revealed the appearance of two exchangeable protons corresponding to NHs at 9.8 and 10.0 ppm, aromatic protons at 7.08–8.34 ppm in addition to cyclohexyl protons at 1.78, 2.9 and 3.0 ppm. ^13^C NMR revealed the appearance of peaks at 168.03 (C_4_ pyrimidine), 163.51 (C_2_ pyrimidine), 153.10 (C = O), 22.27, 22.84, 25.45, 25.94 (cyclohexyl protons). Quantification was performed with multiple reaction monitoring (MRM) by using gas collision-induced dissociation and the following ion transitions: M/z 465.12/252.2 and 519.1/324.1 for sorafinib and **KM6**, respectively, with the cone voltages all set at 45 V and the collision energy at 35 eV. Typical chromatograms for detection of Sorafinib and **KM6** are displayed in Supplementary Data SI. They were detected at retention times 0.98 and 11.1 min. Calibration curve was linear (*r*^2^ ≥ 0.99).

A solution of the urea derivative (1) (0.147 g, 1 mmol) and caesium carbonate (0.171 g, 2 mmol) in dry acetonitrile (10 ml) was stirred for 1 h at room temperature. The 4-chloro thieno[2,3-*d*]pyrimidine derivative (4) (0.1 g, 1 mmol) was added slowly and the reaction mixture was heated for 6 h at 55–60 °C. The solvent was removed under reduced pressure and the residue was stirred with cold 4% NaOH solution (20 ml, 1 M) for 1 h. The solid product was filtered and purified with column chromatography (eluent hexane: ethyl acetate 90:10 to 85:15) to afford **KM6** m.p. 250–253 °C. MS: (Mwt 518.94) (ES mass) (rel. int.) 519.08, (M^+^, 32%), ^1^H-NMR (400 MHz, DMSO-d_6_) δ: 10.08 (broad, 1H exchangeable NH), 9.83 (broad, 1H, exchangeable NH), 8.47 (s, 1H, pyrimidine), 8:16 (s, 1H, Ar-H), 7.55–7.73 (m, 2H, ArH), 7.21 (d, 2H, *J* = 8 Hz, Ar), 7.19 (d, 2H, *J* = 8 Hz, ArH), 3.02–3.33 (m, 2H, cyclohexyl), 2.8–2.9 (m, 2H cyclohexyl), 1.87–1.91 (m, 4H, cyclohexyl). ^13 ^C-NMR (100 MHz, DMSO-d_6_) δ: 168.0, 163.5, 153.1, 152.5, 147.1, 140.2, 137.5, 136.0, 132.4, 127.4, 127.0 (2 C), 126.0 (q, ^2^*J*_CF_ −30.7 Hz), 123.3, 122.6 (q, ^3^*J*_CF_ = 4.1 Hz), 120.0 (2 C),119.0 118.8, 117.1, 25.9, 25.4, 22.8, 22.2, Elemental Analysis: Calcd for C_24_H_18_ClF_3_N_4_O_2_S; C 55.55; H 3.50; N 10.80 Found; C 55.41; H 3.53; N 10.63 (Supplementary data SI, SII, SIII, SIV).

***KM6****:*[1–(4-Chloro-3-trifluoromethylphenyl)-3–(4-((5,6,7,8-tetrahydrobenzo[4,5]thieno[2,3-*d*]pyrimidin-4-yl)oxy)phenyl)urea] was prepared as stock solution at concentration 10 mM and was kept at −20 °C. Immediately before use, it was serially diluted in RPMI 1640 to yield a concentration range of 2–32 µM.

#### Cell lines

Human BC cell lines: MCF7, T47D and MDA-MB-231were obtained from the American Type Culture Collection (ATCC, Minnesota, USA) and were maintained at National Cancer Institute (NCI), Cairo, Egypt in RPMI1640 medium containing 10% foetal bovine serum and 1% penicillin-streptomycin. LCC2 was a generous gift from Dr Robert Clarke (office of technology commercialisation, Georgetown University, USA) and was cultured in EMEM medium containing 5% charcoal foetal bovine serum and 1% penicillin–streptomycin. All cell lines were routinely incubated in 5% CO_2_ in a humidified atmosphere at 37 °C.

### Biological evaluation

#### Cytotoxicity assay

The antitumor activities of **KM6** and TAM on BC cells were evaluated by sulphorhodamine-B (SRB) assay^32^. Briefly, cells were seeded at a density of 3 × 10^3^ cells/well in 96-well microtiter plates. They were left to attach for 24 h before incubation with drugs. Next, cells were treated with different concentrations 2–32 µM of **KM6**, 2 − 14 µM of TAM for MCF7, T47D and MDA-MB-231 cells and by 5–80 µM of TAM for LCC2. For each concentration, three wells were used and incubation was continued for 48 h. DMSO was used as control vehicle (1% v/v). At the end of incubation, cells were fixed with 20% trichloroacetic acid, stained with 0.4% SRB dye. The optical density (O.D.) of each well was measured spectrophotometrically at 570 nm using ELISA microplate reader (TECAN sunrise™, Germany). The percentage of cell survival was calculated as follows: Survival fraction = O.D. (treated cells)/O.D. (control cells). The IC_50_ (concentration that produce 50% of cell growth inhibition) value of each drug was calculated using sigmoidal dose–response curve-fitting models (Graph Pad Prizm software, version 5).

In all the mechanistic experiments in this study, we used the IC_50_ concentrations of TAM and **KM6** in both cell lines. Therefore, in MCF-7, 11 µM of TAM and 6.4 µM of **KM6** were used, while in LCC2 the concentrations used were 67.6 µM of TAM and 3.6 µM of **KM6**.

#### Molecular docking study

To get insights of the binding mode, interaction and orientation of **KM6** into the ATP binding site of VEGFR-2 kinase enzyme, molecular docking simulation study was performed using C-Docker protocol in Discovery Studio 2.5 Software to rationalise the VEGFR-2 inhibitory activity of **KM6**.

To achieve this, the crystal structure of VEGFR-2 kinase enzyme, complexed with sorafenib with PDB code **(4ASD)**, revealed that the substituted terminal phenyl ring lied in a deep extended hydrophobic pocket formed by the movement of Phe1047 residue of the “DFG” motif to induce the “DFG-out” conformation (Figure 2(A)).

Validation of the C-Docker protocol used was performed by redocking the lead compound (sorafenib) in the VEGFR-2 kinase active site and aligning the lead compound's bioactive X-ray conformer with the best-fitted pose acquired from the docking run. The alignment displayed good coexistence between both posed with RMSD = 0.95A°, demonstrating the capability of the used docking protocol to fetch valid docking poses.

To execute the docking protocol, the protein structure was prepared through protein preparation protocol that is integrated into the software. Water molecules were preserved due to their role in VEGFR-2/ligand interaction. The protein structure was also minimised via SMART minimiser algorithm. Afterwards, binding pocket and the surrounding amino acid residues were identified. Compound **(KM6)** was prepared using ligand preparation protocol of Accelry’s Discovery Studio. Finally, docking process was done using C-Docker software in the interface of Accelry’s discovery studio 2.5. Ten docking poses of the docked compound **(KM6)** were generated and examined carefully for selecting the best binding mode similar to sorafenib in the VEGFR-2 binding pocket.

**KM6** was shown to verify the essential key interactions, known for type II VEGFR-2 inhibitors and comparable to sorafenib. Thus, the thieno[2,3-*d*]pyrimidine moiety occupied the flat aromatic hinge region and a hydrogen bond between N1- nitrogen and the main chain NH group of Cys919 residue was observed. In addition, the urea tail was engaged in three key hydrogen bonds with Glu885 and Asp1046 residues. Finally, a Pi-cation interaction with Lys868 residue, as well as a Pi-sigma interaction with Leu 840 residue were established (Figure 2(B)). Its c-Docker interaction energy was estimated to be equal to −48.5 kcal/mol which is comparable to sorafenib −50.7 kcal/mol.

#### Assessment of VEGF kinase activity

The *in vitro* enzyme inhibition determination for **KM6** was carried out in BPS Bioscience Corporation, San Diego, CA (www.bpsbioscience.com). The VEGFR tyrosine kinase activity at single dose concentration of 10 µM was performed, where VEGFR2 (KDR) (BPS#40301) served as the enzyme source and Poly (Glu, Tyr) sodium salt, (4:1, Glu:Tyr) (Sigma#P7244) served as the standardised substrate. Kinase-Glo Plus Luminescence kinase assay kit (Promega, Madison, USA) was used according to the manufacturer’s instructions.

#### Assessment of VEGF level

MCF-7 and LCC2 cells were plated in 24 well plates with 2.5 × 10^4^ cells/well. After treatment with drugs, the culture medium was aspirated and centrifuged at 10,000 rpm at 4 °C for 10 min and the resultant supernatant was used for determination of VEGF by an ELISA Human VEGF ELISA Kit (Koma Biotech Inc, Seoul, Korea, Catalog No. K0331132) in accordance with the manufacturer’s instructions. The amount of VEGF was expressed as pg/ml. Level of VEGF was determined from calibration curve and the percentage of VEGF was calculated as follows:

Level of VEGF = (Level of treated cells/level of control cells) * 100

The level of VEGF was calculated using sigmoidal dose–response curve-fitting models (GraphPad, Prizm5 software incorporated).

#### Determination of malondialdehyde (MDA) content

Lipid peroxidation products were quantified by measuring MDA level in cell culture lysate of control and treated cells using lipid peroxidation (MDA) Assay Kit (Sigma Aldrich Chemical Co., St. Louis, Catalog No. MAK085) following the manufacturer’s instructions. The absorbance was determined at 532 nm using a spectrophotometer (Spectronic, Milton Roy Co.). The MDA concentration was calculated using sigmoidal dose–response curve-fitting models (GraphPad, Prizm5 software incorporated).

The amount of MDA present in the samples was determined from the standard curve.

C =(Sa/Sv) * D

Sa = Amount of MDA in unknown sample (nmole) from standard curve

Sv = Sample volume (µl) added into the wells

*C* = Concentration of MDA in sample

*D* = Sample dilution factor.

#### Determination of superoxide dismutase (SOD)

The activity of the antioxidative enzyme SOD was determined by SOD determination kit (Sigma Aldrich Chemical Co., St. Louis, Cat no 19160) in cell culture lysate of control and treated cells by following the manufacturer’s instructions. SOD level was calculated relative to the corresponding protein content. The absorbance of the supernatant was determined at 450 nm using a spectrophotometer (Spectronic, Milton Roy Co.). The SOD activity (inhibition rate %) was determined using the equation:

SOD activity (inhibition rate %) = {[(Ablank 1 − Ablank 3) – (Asample − Ablank 2)]/(Ablank 1 − Ablank 3)} × 100

#### Determination of reduced glutathione (GSH) content

Reduced glutathione was determined adopting Ellman’s method^33^. MCF7 and LCC2 cells were harvested, protein was precipitated with trichloroacetic acid and Ellman’s reagent [5,5-dithiobis-(2-nitrobenzoic acid)] (Sigma Aldrich Chemical Co, St. Louis) was added to supernatant. GSH The absorbance was determined at 405 nm using spectrophotometer (Spectronic, Milton Roy Co.). GSH content was calculated from standard calibration curve and was expressed as µM using sigmoidal dose–response curve-fitting models (GraphPad, Prizm5 software incorporated).

#### Determination of nitric oxide (NO) content

Nitric oxide was determined in culture media of the control and treated cells according to Miranda et al.^34^ Briefly, 0.5 ml cold absolute ethanol was added to 250 µl culture media then left for 48 h at 4 °C to attain complete protein precipitation followed by centrifugation at 13,000 rpm for 1 h using cooling centrifuge. 100 µl of nitrate standard solution were serially diluted in duplicate in a 96-well microplate. Then, 100 µl of vanadium chloride were added to each well rapidly followed by 50 µl sulphanilamide and 50 µl n-(1-naphthyl) ethylene diamine in 2 N hydrochloric acid. The absorbance at 540 nm was measured following incubation period of 30 min spectrophotometrically using an ELISA microplate reader (TECAN SunriseTM, Germany). The level of total nitrite/nitrate was expressed as µM supernatant media and determined using standard curve.

#### Assay of caspases 3, 8 and 9 activity

Activities of caspases 3, 8 and 9 were measured spectrophotometrically at 450 nm in cell lysate using Colorimetric Assay Kits (Biovision, CA) (Cat numbers K106-25, K113-25 and K119-25, respectively) following the manufacturer’s instructions. Percentage change in caspase activity was determined by comparing the results with the level of the untreated control. The experiment was carried out three independent times.

#### Determination of glucose uptake in cell culture media

In the cell culture medium of the control and treated cells, the concentration of glucose was determined using Colorimetric Assay Kit (Randox, County Antrim, UK) (Cat No.GL 346) following the manufacturer’s instructions. The absorbance was recorded at a wavelength of 500 nm using a spectrophotometer (Spectronic, Milton Roy Co.). Glucose conc. (mg/dl) = $\frac{\text{A sample }}{\text{ A standard}}$ × concentration of standard

#### Determination of lactate dehydrogenase (LDH) in cell culture media and cell lysate

In this study, LDH was measured in the media for the detection of cytotoxicity (necrotic cell death). It was also measured intracellularly after cell lysis as a marker for glycolytic pathway. Thus, MCF-7 and LCC2 cells were plated in 24 well plates with 2.5 × 10^4^ cells/well. After treatment with drugs, the medium was aspirated, centrifuged at 10,000 rpm for 10 min at 4 °C to remove any dead cells and was used for necrosis experiments. Cell pellets were lysed using protein lysis buffer and used for the determination of LDH. UV assay kit was used following manufacturer instructions (Randox, County Antrim, UK, Cat no. LD 401). The activity was calculated relative to the corresponding protein content. The absorbance was recorded at 340 nm using a spectrophotometer (Spectronic, Milton Roy Co.).

LDH activity (U/L) = 4921 × ( 340 nm/min)

#### Determination of prostaglandin E2 (PGE2)

The concentration of PGE2 in cell culture media of the control and treated cells was determined using the PGE2 Enzyme Immunoassay Kit (R&D systems Inc, Minneapolis, (Cat No. PKGE004B) following the manufacturer’s instructions. The concentration of PGE_2_ was calculated in comparison with a plotted standard curve and was expressed as pg/ml.

#### Real-time polymerase chain reaction

Total RNA was extracted from cells using RNeasy Mini Kit (Qiagen, Valencia, Netherlands). cDNA was prepared from RNA (200 ng) in a 20 µl reaction using High capacity cDNA archive kit (Applied Biosystem, CA). Real-time PCR of GAPDH, COX-2, IL-1β, IL-6, MMP2, MMP-9, Survivin, AKT and Ki-67 were performed in triplicate on an ABI 7500 fast real-time PCR System using GoTaq PCR master mix (Promega, Madison). Fast amplification parameters were as follows: an initial denaturation step at 95 °C for 10 min, followed by 45 cycles of denaturing at 95 °C for 15 s, annealing at 60 °C for 1 min, then extension at 72 °C. All primers used in this study were purchased from Invitrogen (CA) (Supplementary Table (SV)). Quantitative analysis of data was performed by using the ΔΔCt method^35^.

The cycle threshold (Ct) was determined automatically.

$$\Delta {\;\text{CT}}_{\left(1\right)}\left(\text{for}\;\text{treated}\;\text{sample}\;\text{for}\;\text{gene}\;\text{of}\;\text{interest}\;\left(x\right)\right)\;={\;\text{CT}}_{\left(X\right)}–{\;\text{CT}}_{\left(\text{GAPDH}\right)}$$

$$\Delta {\;\text{CT}}_{\left(2\right)}\left(\text{for}\;\text{Control}\;\text{sample}\;\text{for}\;\text{gene}\;\text{of}\;\text{interest}\;\left(x\right)\right)\;={\;\text{CT}}_{\left(X\right)}–{\;\text{CT}}_{\left(\text{GAPDH}\right)}$$

ΔΔ CT = Δ CT(1) − Δ CT(2)

$$2^{-\Delta \Delta \;\text{CT}}=\;\text{relative}\;\text{expression}\;=\Delta \Delta \;\text{CT}\;=\;\Delta {\;\text{CT}}_{\left(1\right)}-\;\Delta {\;\text{CT}}_{\left(2\right)}$$

#### Agarose gel electrophoresis

Agarose gel electrophoresis was used for assessing BAX, BCL2 and P53. Total RNA was extracted from cells using RNeasy Mini Kit (Qiagen, Valencia, Netherlands). cDNA was prepared from RNA (200 ng) in a 20 µl reaction using high capacity cDNA archive kit (Applied Biosystem, CA). mRNA expression was measured by gel electrophoresis and β-actin was used as internal control. Primer sequences are displayed in Supplementary Table (SVI).

#### Determination of protein concentration

After incubation of control and treated cells for the specified times, media were collected and stored at −80 °C and cells were harvested by trypsinization then lysed with RIPA lysis buffer (25 mM Tris HCL pH 7.6, 150 mM NaCl, 1% triton X-100, 1% sodium deoxycholate and 0.1% SDS) containing protease inhibitors. Protein concentrations in media and cell lysate were determined by Bradford Assay kit (Pierce, Rockford, IL). All experiments were run in triplicate.

#### Western blot analysis

After treatment of cells, cell pellet was resuspended in cold PBS and subjected to centrifugation, the pellets were collected. The cell pellets were lysed by resuspending in 1 ml complete RIPA buffer containing protease/phosphatase inhibitor cocktail and placed on ice for 30 min till complete lysis. The lysate in RIPA buffer was transferred to an Eppendorf and centrifuged for 15 min at 13,000 rpm at 4 °C. Extracted proteins were seperated by SDS-PAGE (12% acrylamide) and blotted onto PVDF membranes. The membranes were probed with IL-6 and β-Actin antibodies for normalisation. Band intensities were quantified using Win Image Studio Lite_5.2.5 software using β-actin as loading control. Data are represented as mean ± SD.

#### Gelatine zymography

MMP-2 and MMP-9 enzymatic activities in media of the control and treated cells were determined by SDS-PAGE gelatine zymography^36^. Gels were incubated for 15 min in renaturation buffer containing 2.5% triton X-100 at room temperature, washed with water and incubated overnight at 37 °C in developing buffer [5 mM CaCl2, 0.05% Brij 35, and 50 mMTris (pH 7.8)]. They were stained with 0.5% coomassie brilliant blue R-250 for 1 h and then destained in a 50% methanol and 10% acetic acid solution. Clear bands represented areas of proteolytic activity. Human recombinant MMP-2 and MMP-9 were loaded separately as positive controls. Gels were scanned using image Scanner III LabScan 6.0. To determine mean intensity of each band (mean pixel), the band densities were measured with Win_Image Studio Lite_5.2.5software. Data are represented as means ± SD.

### Statistical analysis

Data are represented as means ± SD. The data were analysed using one-way analysis of variance (ANOVA) test. To assess the significance of differences, the Tukey post hoc test was used. *P* values less than 0.05 were statistically significant. Graphs were performed using Prism software programme (graph pad prism software, version 5, CA) and analysis of data was performed using GraphPad InStat, version 5.

## Results and discussion

### KM6 shows cytotoxicity to different BC cell lines including resistant cells

**Figure 1.**
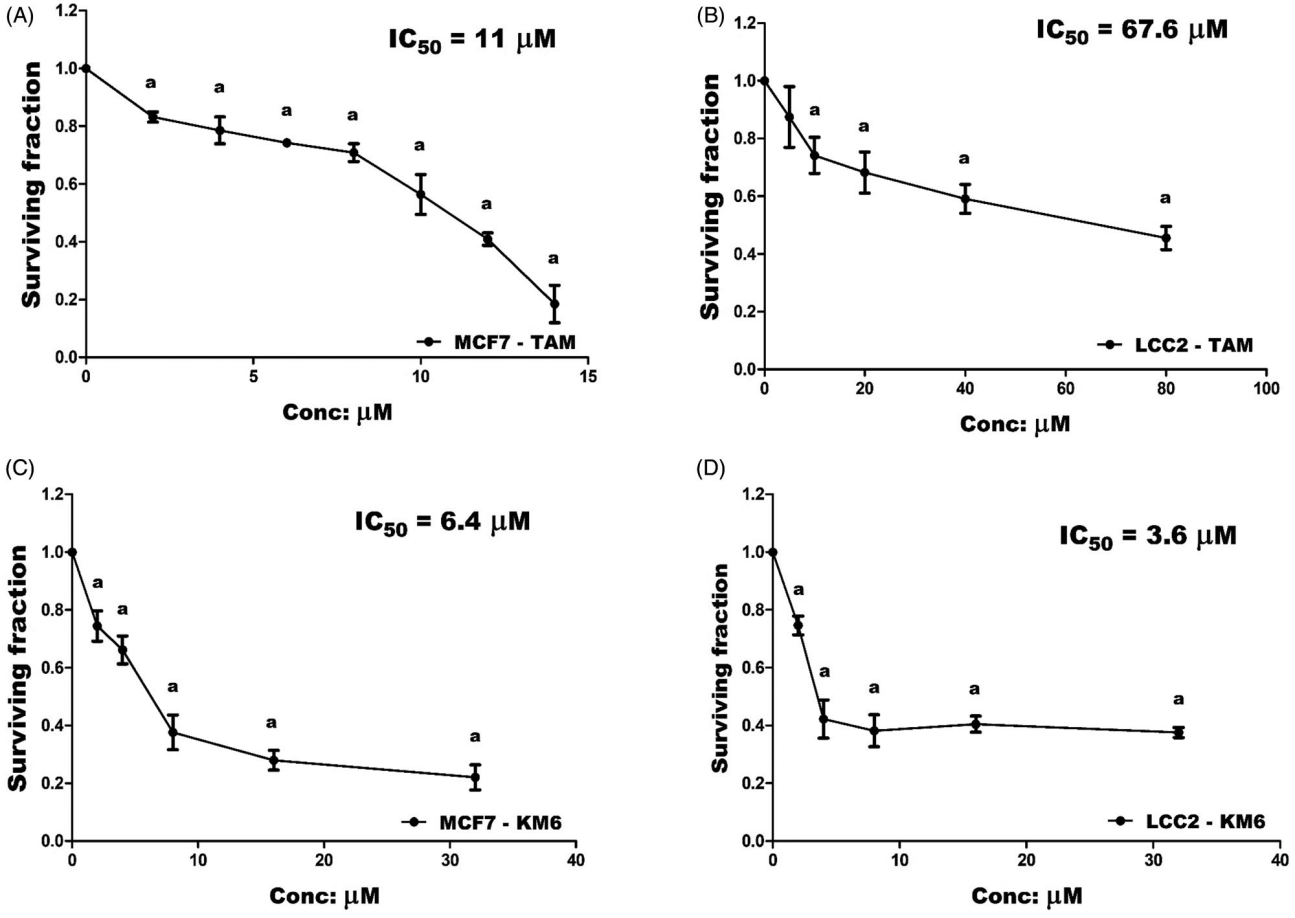
Cytotoxicity of TAM and **KM6** on BC cell lines: (A, B) Effect of TAM on MCF7 and LCC2, respectively. (C, D) Effect of **KM6** on MCF7 and LCC2, respectively, by sulphorhodamine assay. Graphs and data analysis were performed using GraphPad InStat, version 5. The data represent means of at least three independent experiments ± SD. ^a^Significantly different from control (*P* < 0.05).

Both TAM and **KM6** were tested for their cytotoxicity against TAM sensitive MCF7 cells and TAM-resistant LCC2 cells. The IC_50_ of TAM in LCC2 was around 6 folds higher than in MCF7 (67.6 ± 0.07 µM *vs* 11 ± 0.05 µM) thus confirming the resistant phenotype of LCC2 cells (Figure 1(A,B)). Interestingly, **KM6** displayed a more remarkable antitumor activity than TAM on both MCF7 (IC_50_ = 6.4 ± 0.05 µM) and LCC2 (IC_50_ = 3.6 ± 0.03 µM) (Figure 1(C,D)).

This was in agreement with the study of Pedersen et al.^14^ who reported preferential growth inhibition of TAM-resistant cell line with the tyrosine kinase inhibitor, sorafenib due to reduction in the total expression of oestrogen receptor α (ER α), phosphorylated ER, FOXA1 and AIB1 leading to re-sensitisation of TAM-resistant cells to TAM. It is noteworthy that **KM6** also demonstrated remarkable cytotoxic activity against other BC cell lines T47D (IC_50_ = 6.9 ± 0.04 µM) and MDA-MB-231 (IC_50_ = 10 ± 0.04 µM) (Supplementary Figure (SVII)). In the subsequent experiments, we aimed to study the different mechanisms whereby **KM6** exerted its cytotoxic activity in MCF-7 and LCC2 cell lines.

### Effect of KM6 on angiogenesis

**Figure 2.**
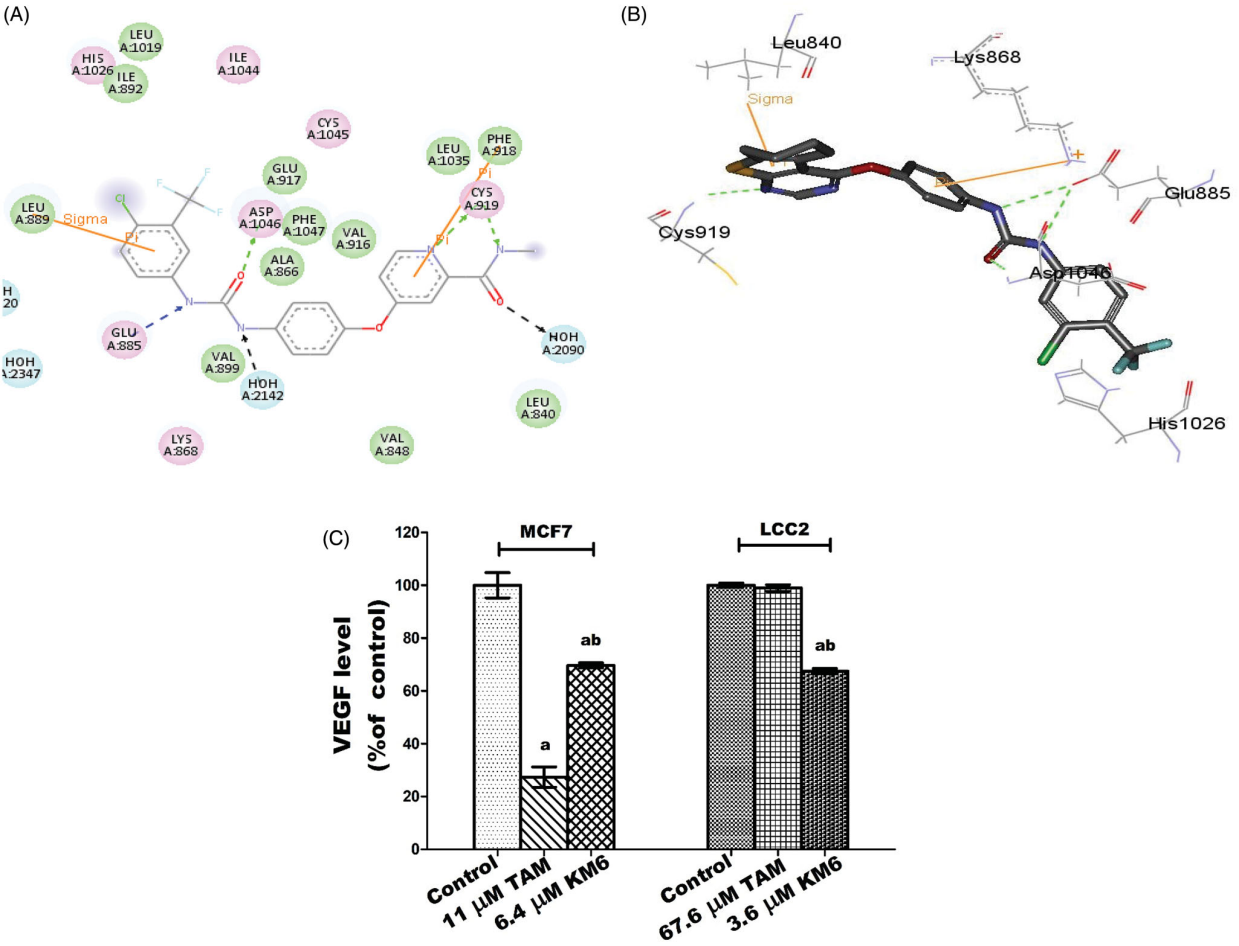
(A) 2D interaction diagram of sorafenib co-crystallized with VEGFR-2 showing two hydrogen bonding with Cys919 (PDB ID: 4ASD) (B) Docking pose of **KM6** into VEGFR-2 kinase enzyme. (C) Effect of TAM and **KM6** on VEGF level in MCF7 and LCC2. Statistical significance of results was analysed using one-way ANOVA followed by Tukey’s multiple comparison test. ^a^Significantly different from control, b from TAM and (*p* < 0.05). TAM was used at IC _50_ (11 µM for MCF7 and 67.6 µM for LCC2). **KM6** was used at IC _50_ (6.4 µM MCF7 and 3.6 µM for LCC2).

**KM6** was evaluated for its ability to inhibit VEGFR-2 tyrosine kinase enzyme activity *in vitro* at a single dose concentration of 10 µM as a measure to suppress angiogenesis. At such concentration, **KM6** exhibited 65% VEGFR-2 kinase inhibition. Moreover, **KM6** decreased VEGF level in both MCF7 and LCC2 culture media by 30.4 and 33%, respectively, compared to their corresponding controls. This is similar to sorafenib and other analogues that is a well-known inhibitor of VEGF in BC^37–39^. However, TAM alone exhibited a more remarkable reduction of VEGF level in MCF7 than **KM6** by 42.3% but no significant effect was observed on VEGF in LCC2 cells (Figure 2(C)). This may be due to the antiestrogenic nature of TAM in BC while oestrogens are known to enhance VEGF expression^40^^,^^41^.

### KM6 downregulates Ki-67 mRNA in both MCF7 and LCC2 cells.

**Figure 3.**
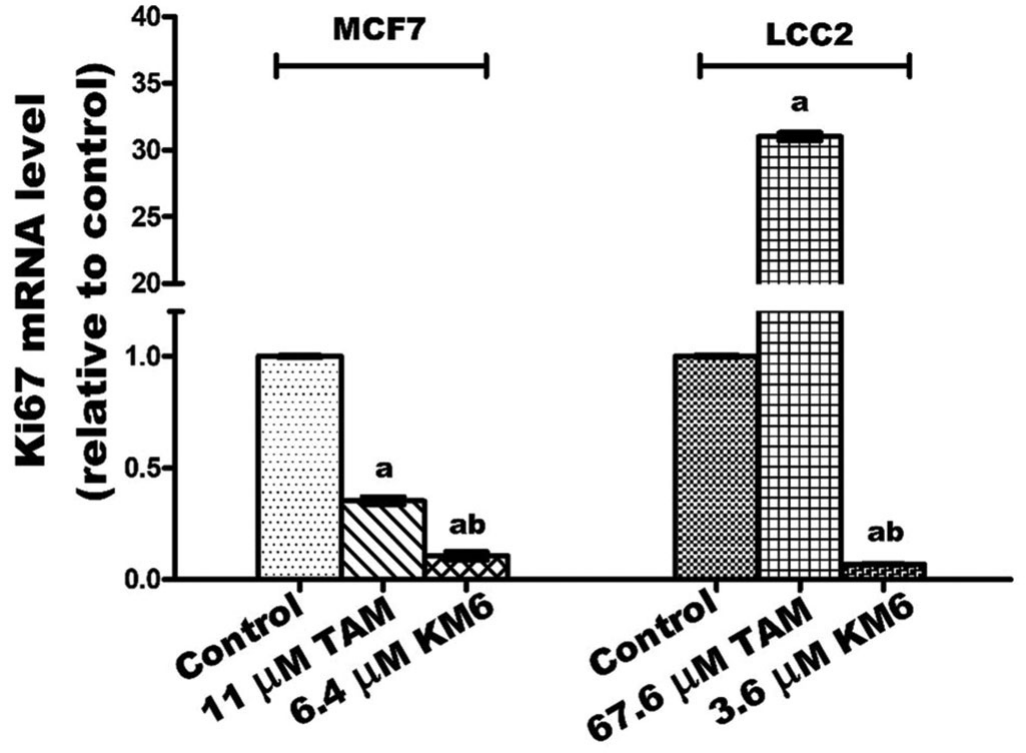
Effect of TAM and **KM6** on Ki-67 mRNA expression in MCF7 and LCC2 by real time RT-PCR. Statistical significance of results was analysed using one-way ANOVA followed by Tukey’s multiple comparison test. ^a^Significantly different from control, b from TAM and (*p* < 0.05). TAM was used at IC_50_ (11 µM for MCF7 and 67.6 µM for LCC2). **KM6** was used at IC_50_ (6.4 µM MCF7 and 3.6 µM for LCC2).

The cell proliferation marker Ki-67 is a nuclear non-histone protein present in all active phases of the cell cycle except in G_O_ phase and is a promising molecular target in the diagnosis of cancer^42^. Previous reports pointed to the role played by Ki-67 in the molecular subtypes of BC^43^ as well as in TAM-resistant BC patients^44^. Additionally, it was reported that sorafenib inhibits liver regeneration in rats through a mechanism involving Ki-67^45^. Our data indicated that TAM downregulated the expression of Ki-67 in MCF7 while it induced a pronounced increase in its expression in LCC2 cells. This effect may be due to the previously reported molecular changes associated with the antagonistic activity of TAM in MCF7, and the hyper-response of oestradiol in TAM-resistant BC cell lines^46^. On the other hand, **KM6** downregulated Ki-67 in both MCF7 and LCC2 (Figure 3).

### KM6 is pro-oxidant in MCF7 but anti-oxidant in LCC2

**Figure 4.**
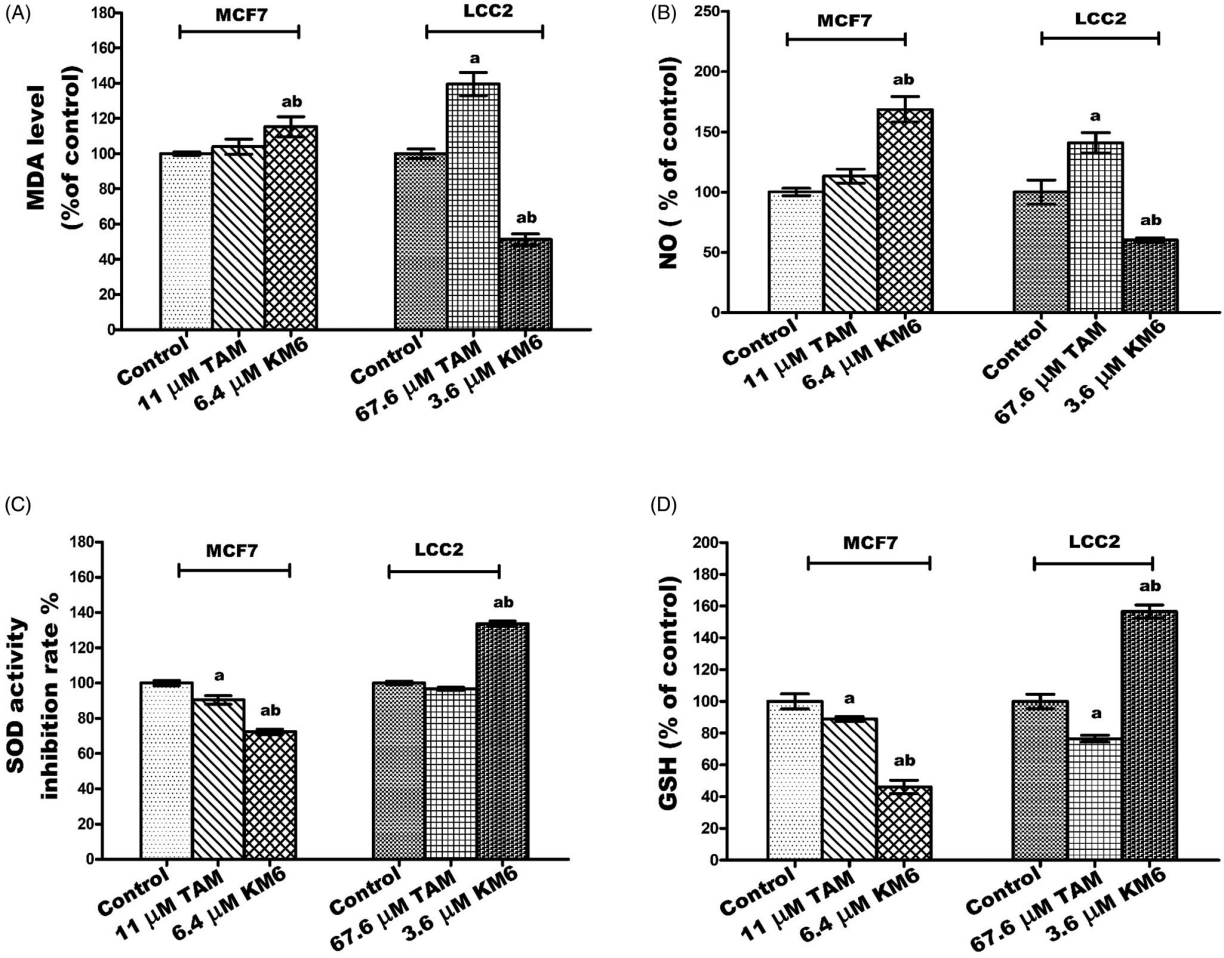
Effect of TAM and **KM6** on oxidative stress markers (A) MDA, (B) NO, (C) SOD and (D) GSH in MCF7 and LCC2. Statistical significance of results was analysed using one-way ANOVA followed by Tukey’s multiple comparison test. ^a^Significantly different from control, b from TAM and (*p* < 0.05). TAM was used at IC 50 (11 µM for MCF7 and 67.6 µM for LCC2). **KM6** was used at IC 50 (6.4 µM MCF7 and 3.6 µM for LCC2).

Cancer cells are characterised by an increase in the rate of reactive oxygen species (ROS) production and an altered redox environment compared to normal cells. Most chemotherapeutics agents raise intracellular levels of ROS, and can alter redox homeostasis of cancer cells^47^. Consequently, **KM6** was tested for its antioxidant activity in both MCF7 and LCC2 cells. **KM6** induced a pro-oxidant state in MCF7 by significantly increasing the MDA and NO by 15.3% and 68%, respectively, accompanied by a significant reduction of SOD by 28% and GSH by 53%. On the other hand, **KM6** had anti-oxidant effect in LCC2 where it significantly increased SOD by 33.6% and GSH by 56.7% while MDA and NO levels were significantly decreased by 48.8% and 39.8%, respectively. Compared to TAM, **KM6** acts as a stronger prooxidant in MCF7 rather than the resistant cells. It's noteworthy that previous reports attributed a great deal of sorafenib cytotoxicity to the generation of reactive oxygen species^48^_._ Thus, it can be speculated that generation of free radicals contributes to the cytotoxicity of **KM6** in MCF7 rather than in LCC2 as in Figure 4(A–D).

### KM6 affects apoptosis in both MCF7 and LCC2

#### KM6 upregulates caspases 3, 8 and 9 in MCF7 and LCC2

The effect of **KM6** on the apoptotic caspases was investigated. This included the caspases involved in the extrinsic pathway (caspase 8), intrinsic pathway (caspase 9) and executioner pathway (caspase 3). The results indicated that in MCF7, **KM6** induced significant increase in caspases 8, 9 and 3 by 53.8, 49 and 76.4%, respectively. A similar pattern was observed in LCC2 where **KM6**-treated cells displayed increase of all the studied caspases. It's observed that the induction of caspases by TAM is more pronounced on MCF7 than **KM6**. However, LCC2 exhibited resistance to the apoptotic effect of TAM. This resistance was not observed in **KM6-**treated cells (Figure 5(A–C)). Previous studies reported that sorafenib exerts antiapoptotic effects through the caspase pathway in prostate cancer^49^.

Figure 5.Effect of TAM and **KM6** on apoptotic and necrotic cell death. (A, B,C) Colorimetric assay of caspases Caspase-8, Caspase-9 and Caspase-3, respectively. (D) mRNA expression of survivin determined by real-time RT-PCR (E) mRNA expression of P53 determined by agarose gel electrophoresis. (F, G) Agarose gel electrophoresis of BAX and BCL-2, respectively. (H) BAX/BCL-2 ratio. (I) Effect of TAM and **KM6** on necrosis as determined by measuring LDH levels in culture media of MCF7 and LCC2. Band analysis was analysed by Win Image Studio Lite_5.2.5software. Statistical significance of results was analysed using one-way ANOVA followed by Tukey’s multiple comparison test. ^a^Significantly different from control, ^b^from TAM and (*p* < 0.05). TAM was used at IC 50 (11 µM for MCF7 and 67.6 µM for LCC2). **KM6** was used at IC 50 (6.4 µM MCF7 and 3.6 µM for LCC2).

**Figure:**
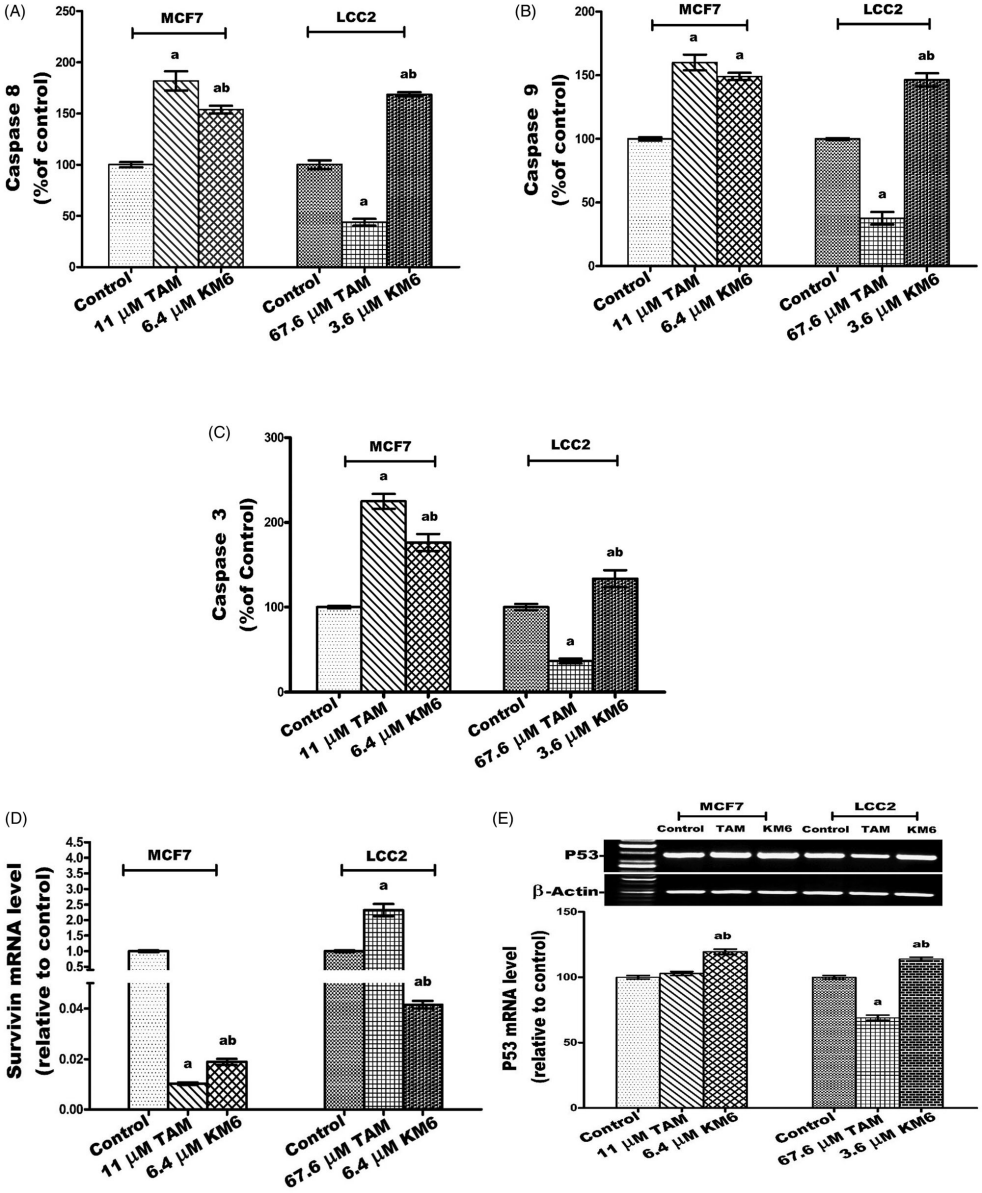

**Figure:**
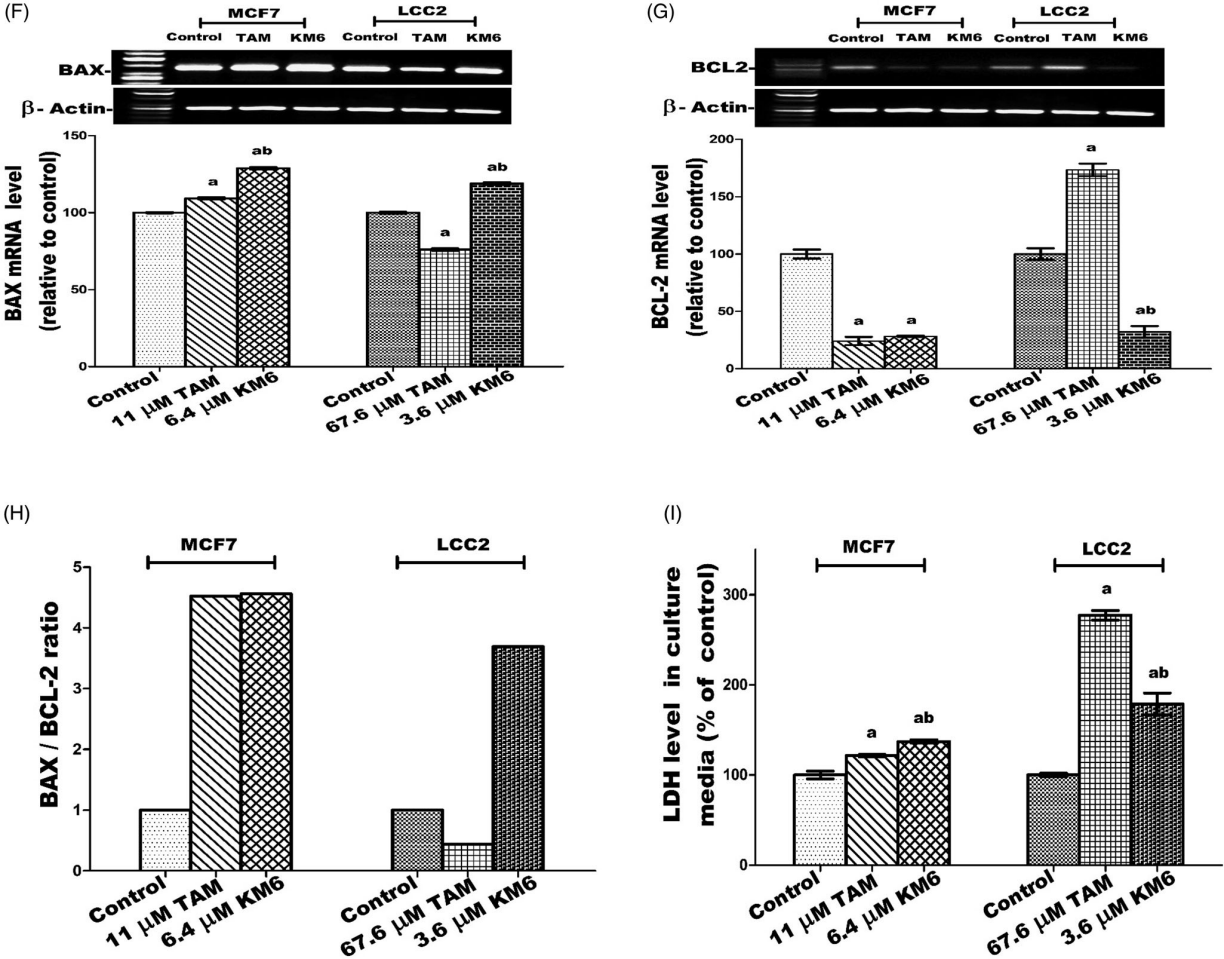

#### KM6 increases P53 mRNA and decreases survivin mRNA in MCF7 and LCC2

The effect of **KM6** on survivin (member of the inhibitor of apoptosis IAP family) was investigated as displayed in Figure 5(D). It was found that survivin was decreased by 53.5 folds compared to control in MCF7 and by 24 folds in LCC2 by **KM6**. TAM also reduced the expression of survivin in MCF7 by 98-fold but increased its expression in LCC2. Concerning the tumour suppressor gene P53 which plays an important role in apoptosis induction^50^, its gene expression was significantly increased by **KM6** by 19.5% in MCF7 and by 13.9% in LCC2 compared to control (Figure 5(E**))**. This upregulation of P53 was previously observed by sorafenib in hepatocellular carcinoma^51^. TAM, on the other hand, did not affect P53 expression in MCF7, which is consistent with previous reports where TAM did not alter P53 protein in MCF7 cells^52^. However, TAM reduced P53 expression in LCC2 by 31% probably due to alteration of apoptotic responses in resistant cells as previously described^53^.

#### KM6 increases BAX/BCL-2 ratio in MCF7 and LCC2

The current study indicated that **KM6** favourably modulates the gene expressions of the apoptotic protein BAX as well as the antiapoptotic proteins BCL-2. As shown in Figure 5(F–H), treatment of MCF7cells by **KM6** resulted in increasing BAX/BCL-2 ratio by 4.56 folds in MCF7 and by 3 folds in LCC2. This was comparable to BAX/BCL-2 ratio produced by TAM in MCF7 (4.52). However, in LCC2, **KM6** was superior to TAM that decreased BAX/BCL-2 ratio to 0.438%. Previous studies demonstrated that sorafenib reduces the antiapoptotic protein Mcl-1, which is a member of BCL-2 family leading to induction of apoptosis in lung cancer cell line^54^ and it sensitises hepatocellular cancer cells to apoptotic stimuli^55^. In addition, TAM downregulated BCL-2 in MCF7 but without affecting BAX^52^, which is different from our study showing slight increase in BAX.

### KM6 increases necrosis in both MCF7 and LCC2

A key sign for necrotic cells is the permeabilization of the plasma membrane that can be detected in tissue culture by quantifying the release of the intracellular enzyme LDH^56^. As depicted in Figure 5(I), the LDH level in both MCF7 and LCC2 increased significantly following treatment by **KM6** by 37.2 and 78%, respectively. TAM also induced necrosis in both cell lines, yet its effect on LCC2 was very pronounced where it increased by 177.1% compared to control group.

### KM6 modulates glucose metabolism through decreasing glucose uptake, LDH and AKT levels in both MCF7 and LCC2

Among the metabolic changes exhibited by tumour cells is an increase in glucose metabolism and glucose dependence. Tumour cells exhibit high levels of glycolysis even in the presence of sufficient oxygen, a phenomenon termed aerobic glycolysis or Warburg effect. This involves increased glucose uptake, glycolytic rates, ATP production, lactate as well as several intermediates and enzymes that benefit rapidly proliferating cells. LDH is a key enzyme in this process that converts most of glucose stores into lactate. As a result, much of glucose metabolites are shifted from simple energy production to the promotion of accelerated cell growth and replication^57^. AKT is a key protein involved in PI3K-AKT signal transduction pathway that promotes survival and growth in response to extracellular signals. Dronamraju et al.^58^ reported that enhanced AKT signalling is associated with activation of various downstream enzymes involved in the glycolytic process.

**Figure 6.**
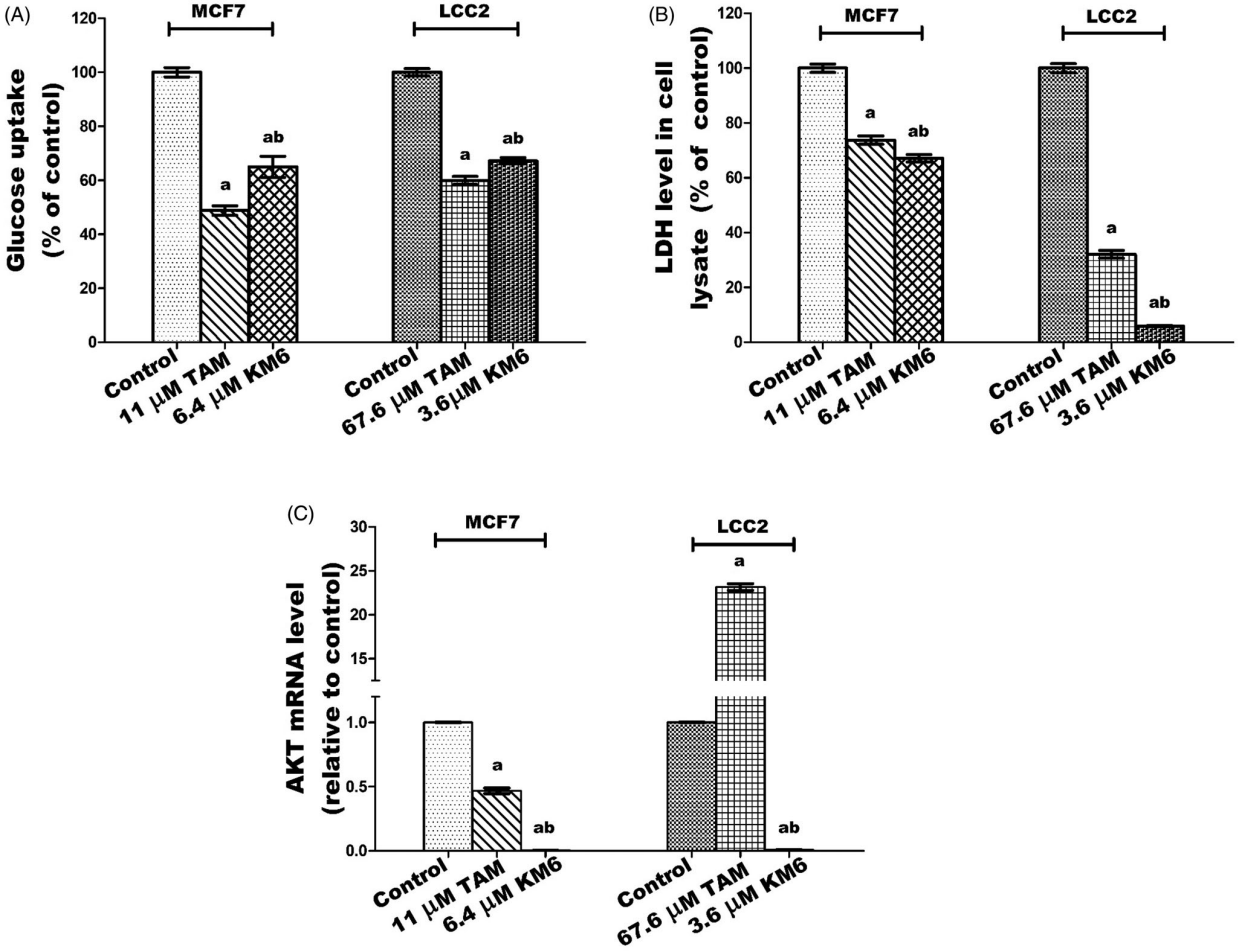
Effect of TAM and **KM6** on glucose metabolism in MCF7 and LCC2. (A) Glucose uptake in media. (B): LDH level in cell lysate. (C) AKT mRNA expression. Statistical significance of results was analysed using one-way ANOVA followed by Tukey’s multiple comparison test. ^a^Significantly different from control, ^b^from TAM and (*p* < 0.05). TAM was used at IC 50 (11 µM for MCF7 and 67.6 µM for LCC2). **KM6** was used at IC 50 (6.4 µM MCF7 and 3.6 µM for LCC2).

In this study, treatment of MCF7 with **KM6** resulted in decline in both glucose uptake and LDH levels by 44 and 32.95%, respectively, relative to their corresponding controls (Figure 6(A,B)). In LCC2, a similar pattern was observed where glucose uptake was reduced by 32.8% and LDH by 94.04%. This is in harmony with previous data indicating that sorafenib curbs glucose utilisation in BC cells through the repression of mTORC1 activity^59^. It is notable that TAM was more effective than **KM6** in reducing glucose uptake in both MCF7 and LCC2 while the opposite was true in case of LDH. Likewise, the results (Figure 6(C**))** showed a significant dramatic decrease in the gene expression of AKT by 232.4 folds in MCF7 and 106.9 folds for LCC2 as compared to control. In addition, a profound decline in AKT expression was observed compared to TAM-treated cells in both cell lines. It’s known that activation of AKT is a mechanism of resistance to sorafenib in hepatocellular carcinoma^60^. In BC, the relation between AKT and sorafenib has not been widely investigated. However, it was demonstrated that sorafenib inhibited cell proliferation and induced apoptosis in a panel of BC cell lines with no correlation to AKT^59^. Meanwhile, activation of PI3K/Akt signalling pathway was found to mediate resistance to sorafenib in hepatocellular carcinoma cells, and the combination of sorafenib and MK-2206, an Akt inhibitor, overcame this resistance at clinical concentrations^60^. Based on our results, **KM6** displays a promising potential in blocking AKT pathway and further studies are required to test its ability to reverse acquired resistance to sorafenib especially in hepatocellular carcinoma.

### KM6 reduces the inflammatory mediators PGE2, COX2, IL-1β and IL6 in both MCF7 and LCC2

The inflammatory environment affects cancer cell behaviour, including cancer formation, invasion and metastasis^61^. Prostaglandin metabolism plays a pivotal role in inflammatory processes and is proven to have a role in carcinogenesis, tumour differentiation and tumour growth in BC. COX-2 is the key involved enzyme while PGE2 acts as a ligand of the G-protein coupled receptors EP2 which enhances VEGF expression associated with tumour neoangiogenesis^62^^,^^63^. Several cytokines also play a role in tumorigenesis. For example, the inflammatory cytokine IL-1β has been reported to induce the expression of markers associated with malignancy in BC cells through Epithelial-Mesenchymal Transition (EMT)^64^. In addition, the effect of IL-6 expression on EMT is mediated through activation of STAT3^65^.

**Figure 7.**
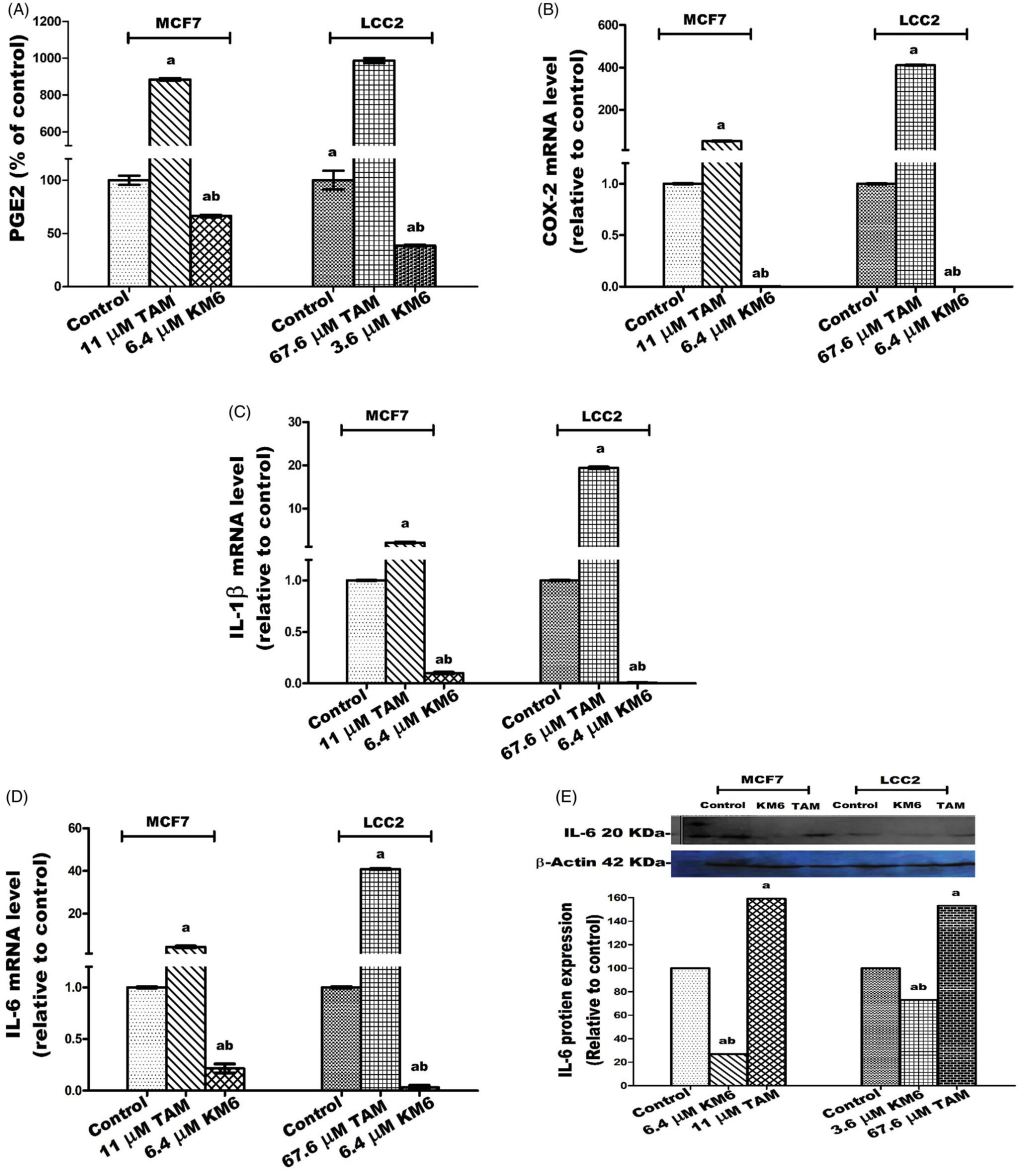
Effect of TAM and **KM6** on inflammatory markers: (A) Effect on PGE2 using ELISA. (B–D) Effect on COX-2, IL-β and IL-6 mRNA expression as determined by real time RT-PCR (E) Effect on protein expression of IL-6 as determined by western blotting. Statistical significance of results was analysed using one-way ANOVA followed by Tukey’s multiple comparison test. ^a^Significantly different from control, ^b^from TAM and (*p* < 0.05). TAM was used at IC 50 (11 µM for MCF7 and 67.6 µM for LCC2). **KM6** was used at IC 50 (6.4 µM MCF7 and 3.6 µM for LCC2).

In this study, the potential effect of **KM6** on several inflammatory mediators (PGE2, COX-2, IL-1β and IL6) has been evaluated (Figure 7(A–E)). The results demonstrated that a significant reduction of all the studied inflammatory markers was detected in both cell lines. As compared to the control group, **KM6** induced significant decrease in PGE2 level by 33.7% in MCF7, and by 61.5% in LCC2. A profound decline in COX-2 mRNA level was detected by **KM6** by 287.9% in MCF7 and by 843.1% in LCC2. **KM6** also induced a reduction in IL-1β and IL-6 by 9.9 and 4.7 folds in MCF7 and by 155.7 and 33.4 folds in LCC2, respectively. Protein expression of IL-6 was also reduced in a similar pattern to mRNA expression following treatment by **KM6**.

The effect of sorafenib on inflammatory markers in BC has not been widely explored. Recently, it was reported that sorafenib improves therapy with alkylating agents by blocking inflammation, invasion and angiogenesis in BC cells^66^. In addition, another study verified that sorafenib inhibits signal transduction in response to exogenous and endogenous cytokines^67^. Thus, in view of our results, the sorafenib analogue **KM6** exerts a promising anti-inflammatory effect in BC.

An interesting observation is an obvious increase mediated by TAM of all the studied inflammatory mediators in both MCF7 and LCC2 and the ability of **KM6** to reverse this inflammatory effect. Further studies are thus required to elucidate the relation between TAM and inflammation in BC as well as the potential use of **KM6** in modulating this effect.

### KM6 downregulates migration and invasion markers MMP-2 and MMP-9 in both MCF7 and LCC2

**Figure 8.**
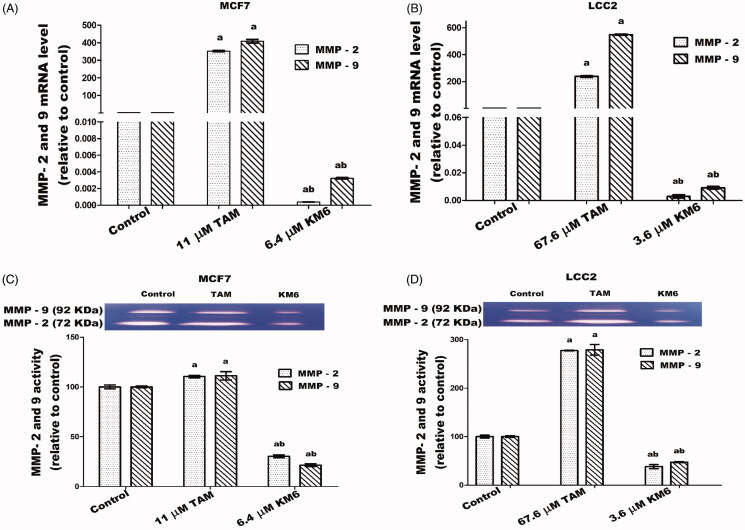
Effects of **KM6** and TAM on invasion and metastatic markers in MCF7 and LCC2. MMP-2 and MMP-9 mRNA expression was determined by real time RT-PCR in MCF7 (A), and LCC2 (B). Also, zymographic activity was determined in both cell lines (C, D). The analysis was done by WinImage Studio Lite_5.2.5software. Statistical significance of results was analysed using one-way ANOVA followed by Tukey’s multiple comparison test. ^a^Significantly different from control, ^b^from TAM and (*p* < 0.05). TAM was used at IC 50 (11 µM for MCF7 and 67.6 µM for LCC2). **KM6** was used at IC 50 (6.4 µM MCF7 and 3.6 µM for LCC2).

The high expression of proteinases such as MMP-9 and MMP-2 in the tumour microenvironment has a role in supporting migration, invasion and metastasis of cancer cells through ECM degradation^68^. In BC tissues, MMP-2 and MMP-9 are highly expressed and directly correlated to lymph node metastasis and tumour staging and thus can be used as reference indices for guiding BC prognosis and treatment^69^. Also, MMP-2 and MMP-9 have been implicated in TAM resistance^70^. Our study shows the downregulation of MMP-2 and MMP-9 gene expression and protein levels by treatment with **KM6** as indicated in Figure 8(A–D). Previous reports about sorafenib similarly indicate that sorafenib suppressed invasion in hepatocellular carcinoma cells through inhibition of matrix metalloproteinase (MMPs) expression^71^. As far as we know, the effect of sorafenib and its derivatives on MMPs in BC has not been widely investigated. However, a previous study concluded that the combination of sorafenib and radiation inhibits BC stem cells partly by reducing MMP-2 expression^72^. On the other hand, as indicated by our data, TAM induced the upregulation of both MMP-2 and MMP-9 in MCF7 and LCC2. This occurred at the level of both gene expression as well as zymographic activity measurements. This is a confirmation of previous data where TAM-induced MMP-2/MMP-9 activities in ER + PR + human BC cells^73^. However, as indicated by our data, their activities were reduced in **KM6**-treated cells, which suggests that this drug may have some antimetastatic activities. Given the multiple pathways of metastasis, further studies are needed to confirm this mechanism.

## Conclusion

From this study it can be concluded that the newly synthesised thieno[2,3-*d*] pyrimidine based urea derivative **KM6** is a potential therapeutic agent for ER + TAM sensitive and resistant BC, providing a basis for development of the compound as a novel anticancer agent. The mechanism of this activity is believed to be through targeting several key enzymes and proteins involved in tumour growth and progression. Through its polypharmacological properties, **KM6** can affect several pathways of angiogenesis, redox activity, apoptosis, inflammation, glucose metabolism and metastasis. Further preclinical, molecular and toxicologic studies are needed to elucidate deeply the main mechanism of action and to determine the optimal dose, route and formulation for administering **KM6**.

## Supplementary Material

Supplemental Material
