# Supplementary material for: Design, synthesis and biological evaluation of a new thieno[2,3-d]pyrimidine-based urea derivative with potential antitumor activity against tamoxifen sensitive and resistant breast cancer cell lines
Source: J Enzyme Inhib Med Chem. 2020 Aug 11;35(1):1641–56. doi: 10.1080/14756366.2020.1804383 (PMC7470147; doi:10.1080/14756366.2020.1804383)
Supplement: Supplemental Material [file IENZ_A_1804383_SM7703.zip › SII.pdf]

Marwa Mohamed_C_KM6

Microanalytical Unit - FOPCU - NMR laboratory
www.pharma.cu.edu.eg dir-mau.fopcu@pharma.cu.edu.eg

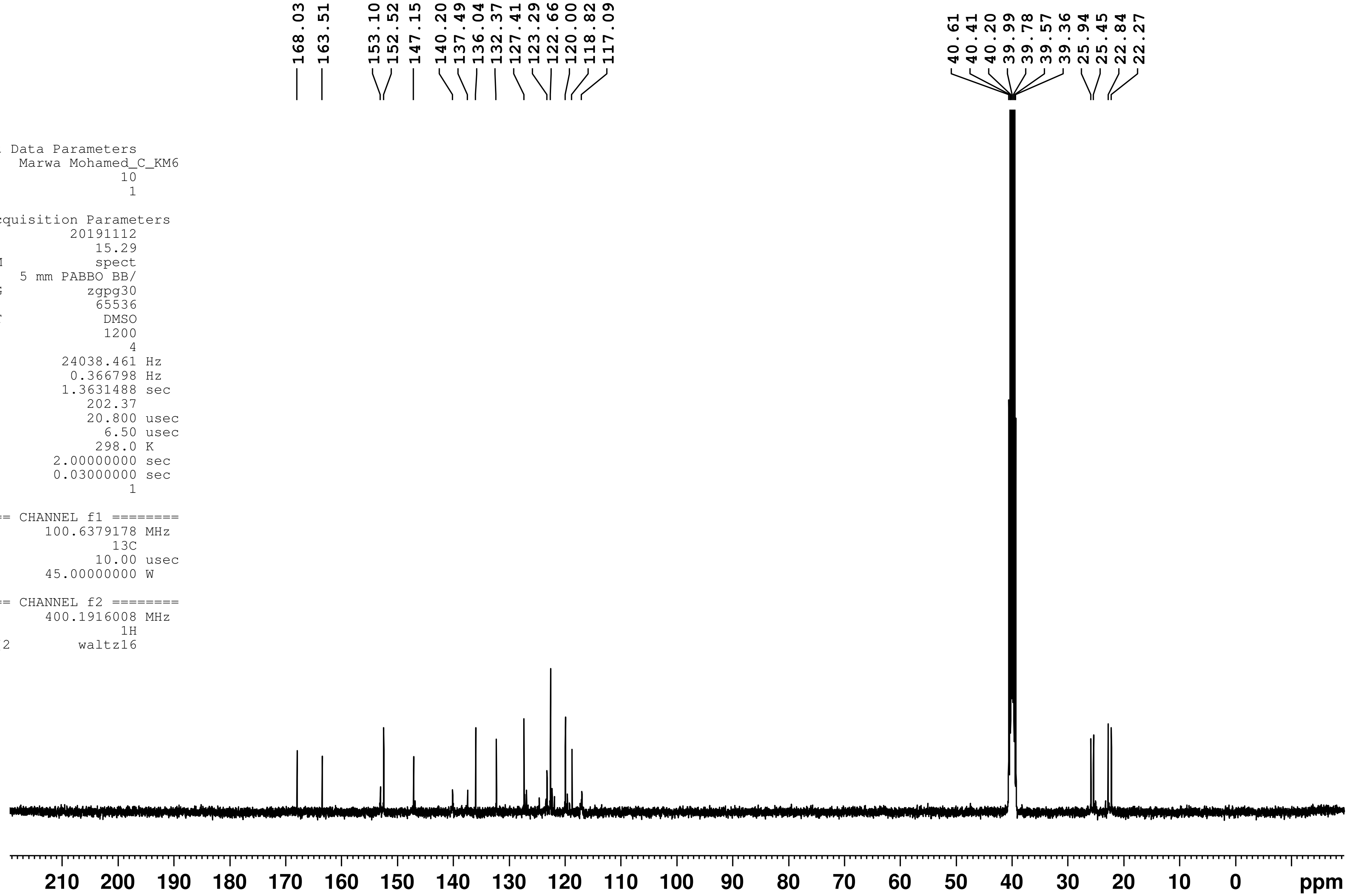


```
Current Data Parameters
NAME      Marwa Mohamed_C_KM6
EXPNO                    10
PROCNO                    1

F2 - Acquisition Parameters
Date_            20191112
Time                15.29
INSTRUM             spect
PROBHD    5 mm PABBO BB/
PULPROG           zgpg30
TD                 65536
SOLVENT             DMSO
NS                  1200
DS                     4
SWH            24038.461 Hz
FIDRES          0.366798 Hz
AQ             1.3631488 sec
RG                202.37
DW                20.800 usec
DE                  6.50 usec
TE                 298.0 K
D1            2.00000000 sec
D11           0.03000000 sec
TD0                    1

======== CHANNEL f1 ========
SFO1         100.6379178 MHz
NUC1                 13C
P1                 10.00 usec
PLW1         45.00000000 W

======== CHANNEL f2 ========
SFO2         400.1916008 MHz
NUC2                  1H
CPDPRG[2         waltz16
```
