# Supplementary material for: Design, synthesis and biological evaluation of a new thieno[2,3-d]pyrimidine-based urea derivative with potential antitumor activity against tamoxifen sensitive and resistant breast cancer cell lines
Source: J Enzyme Inhib Med Chem. 2020 Aug 11;35(1):1641–56. doi: 10.1080/14756366.2020.1804383 (PMC7470147; doi:10.1080/14756366.2020.1804383)
Supplement: Supplemental Material [file IENZ_A_1804383_SM7703.zip › SIII.pdf]

Marwa Mohamed_H_KM6

Microanalytical Unit - FOPCU - NMR laboratory
www.pharma.cu.edu.eg dir-mau.fopcu@pharma.cu.edu.eg

BRUKER

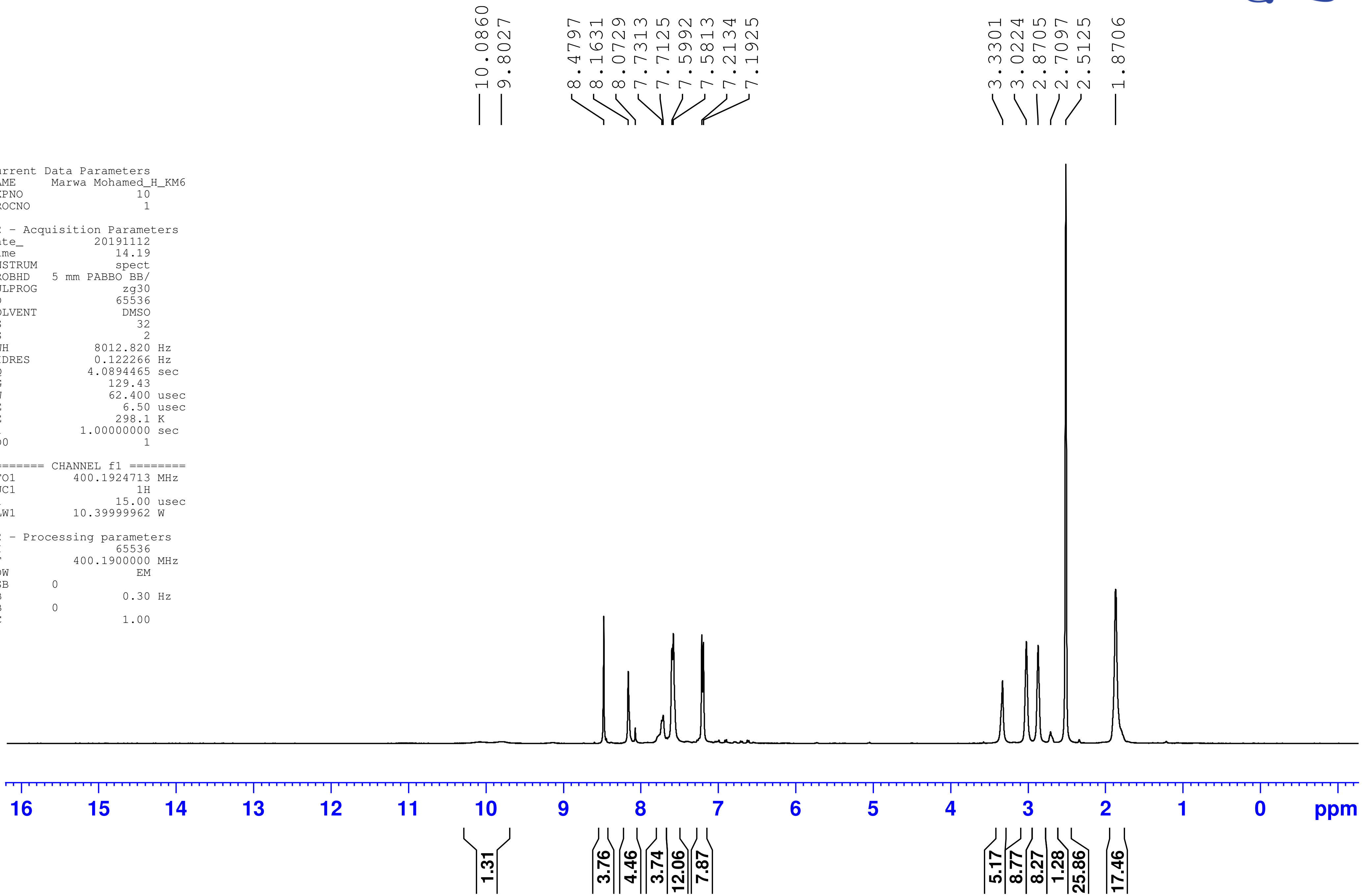


```
Current Data Parameters
NAME      Marwa Mohamed_H_KM6
EXPNO                   10
PROCNO                   1

F2 - Acquisition Parameters
Date_             20191112
Time                 14.19
INSTRUM              spect
PROBHD    5 mm PABBO BB/
PULPROG               zg30
TD                   65536
SOLVENT               DMSO
NS                      32
DS                       2
SWH               8012.820 Hz
FIDRES            0.122266 Hz
AQ               4.0894465 sec
RG                  129.43
DW                  62.400 usec
DE                    6.50 usec
TE                   298.1 K
D1              1.00000000 sec
TD0                      1

======== CHANNEL f1 ========
SFO1           400.1924713 MHz
NUC1                    1H
P1                   15.00 usec
PLW1           10.39999962 W

F2 - Processing parameters
SI                   65536
SF             400.1900000 MHz
WDW                     EM
SSB       0
LB                    0.30 Hz
GB        0
PC                    1.00
```
