# Supplementary material for: Design, synthesis and biological evaluation of a new thieno[2,3-d]pyrimidine-based urea derivative with potential antitumor activity against tamoxifen sensitive and resistant breast cancer cell lines
Source: J Enzyme Inhib Med Chem. 2020 Aug 11;35(1):1641–56. doi: 10.1080/14756366.2020.1804383 (PMC7470147; doi:10.1080/14756366.2020.1804383)

Marwa Mohamed_H_KM6_D2O

Microanalytical Unit - FOPCU - NMR laboratory
www.pharma.cu.edu.eg dir-mau.fopcu@pharma.cu.edu.eg

```
Current Data Parameters
NAME      Marwa Mohamed_H_KM6_D2O
EXPNO                    10
PROCNO                    1

F2 - Acquisition Parameters
Date_            20191113
Time                12.45
INSTRUM             spect
PROBHD    5 mm PABBO BB/
PULPROG              zg30
TD                  65536
SOLVENT              DMSO
NS                     32
DS                      2
SWH              8012.820 Hz
FIDRES           0.122266 Hz
AQ              4.0894465 sec
RG                 129.43
DW                 62.400 usec
DE                   6.50 usec
TE                  298.0 K
D1             1.00000000 sec
TD0                     1

======== CHANNEL f1 ========
SFO1          400.1924713 MHz
NUC1                   1H
P1                  15.00 usec
PLW1          10.39999962 W

F2 - Processing parameters
SI                  65536
SF            400.1900000 MHz
WDW                    EM
SSB       0
LB                   0.30 Hz
GB        0
PC                   1.00
```

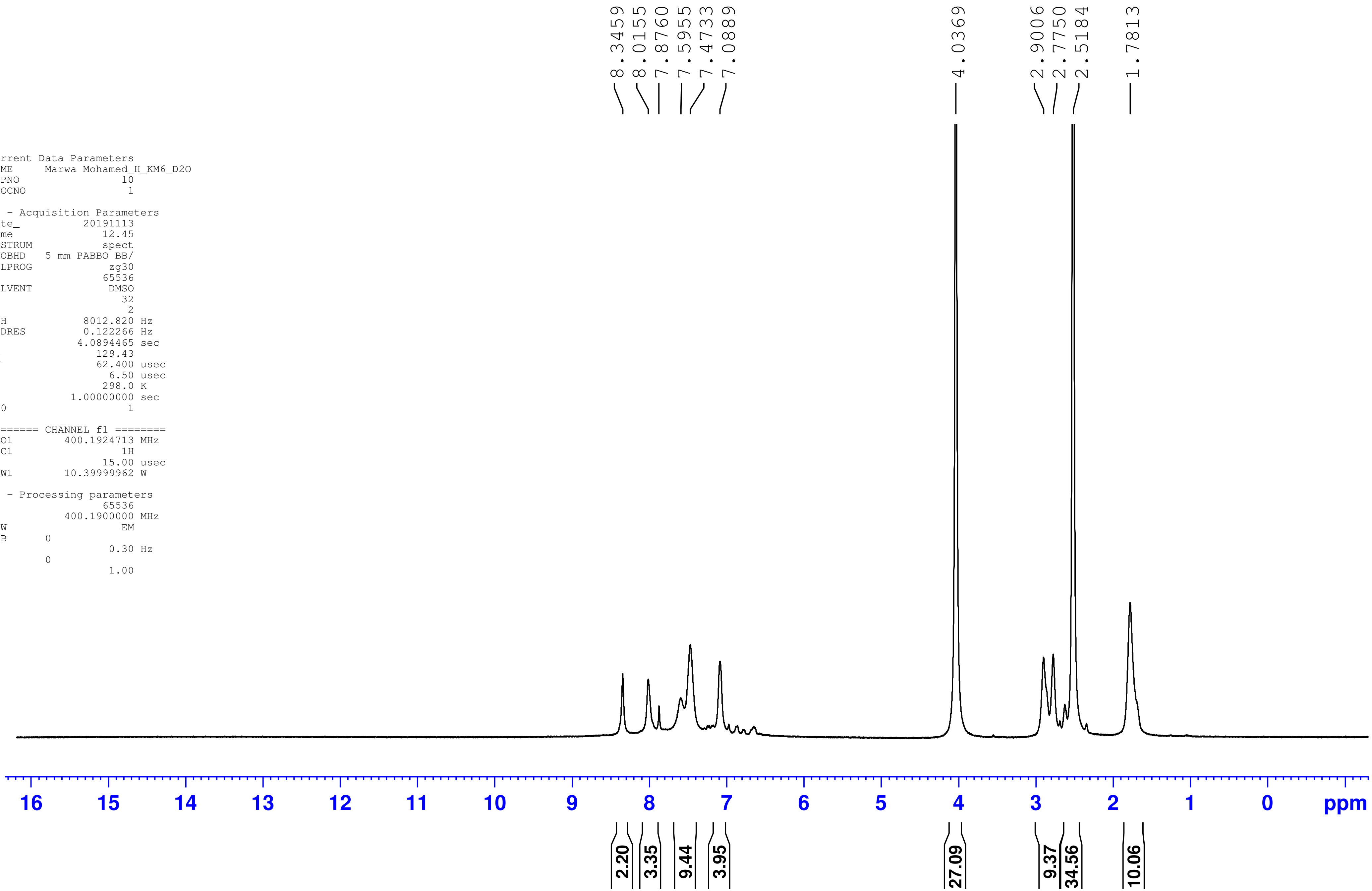

Supplement: Supplemental Material [file IENZ_A_1804383_SM7703.zip › SIV.pdf]
