# Supplementary material for: Design, synthesis and biological evaluation of a new thieno[2,3-d]pyrimidine-based urea derivative with potential antitumor activity against tamoxifen sensitive and resistant breast cancer cell lines
Source: J Enzyme Inhib Med Chem. 2020 Aug 11;35(1):1641–56. doi: 10.1080/14756366.2020.1804383 (PMC7470147; doi:10.1080/14756366.2020.1804383)
Supplement: Supplemental Material [file IENZ_A_1804383_SM7703.zip › SV.pptx]

## Slide 1
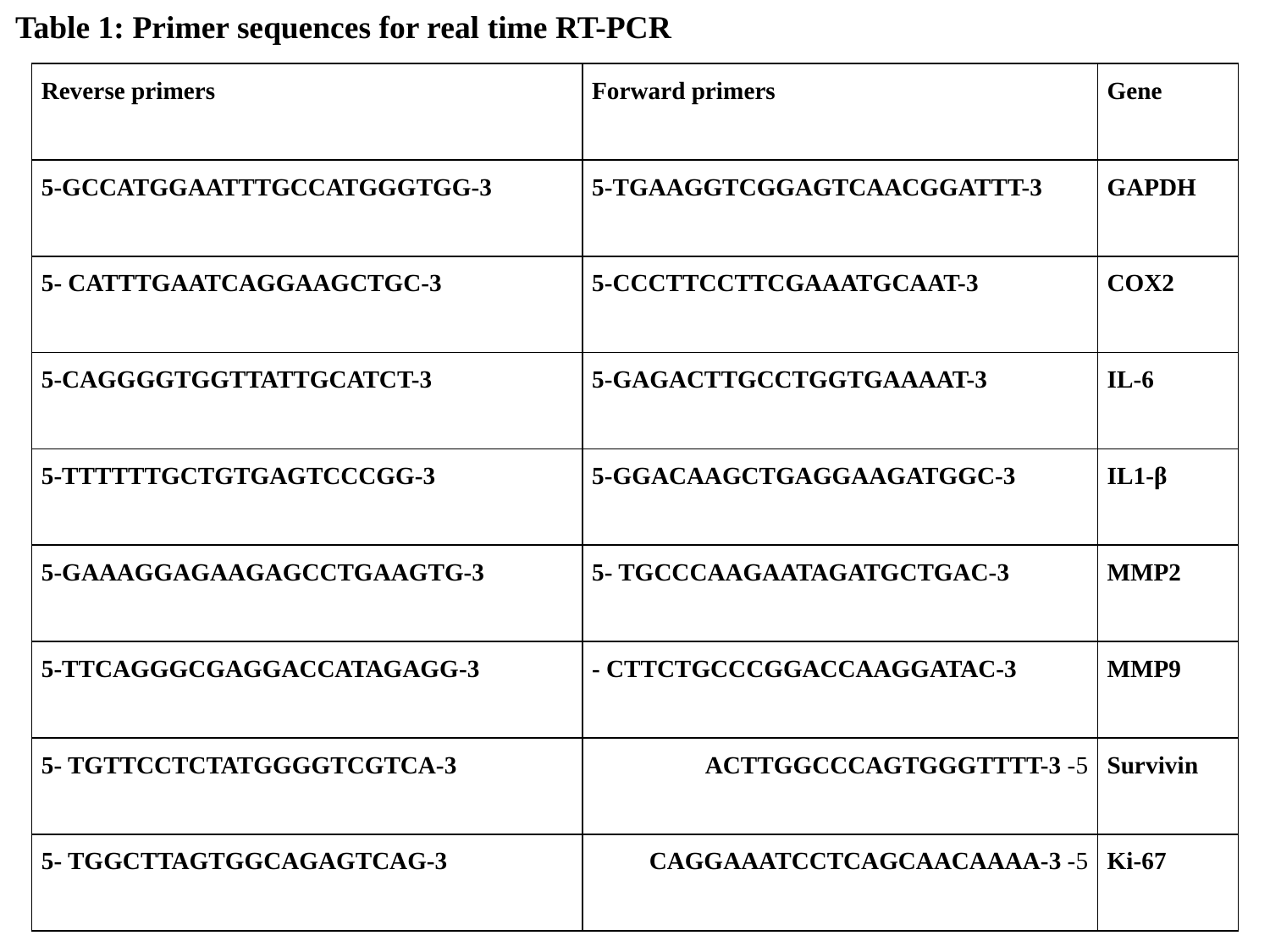

Table 1: Primer sequences for real time RT-PCR
| Reverse primers | Forward primers | Gene |
| --- | --- | --- |
| 5-GCCATGGAATTTGCCATGGGTGG-3 | 5-TGAAGGTCGGAGTCAACGGATTT-3 | GAPDH |
| 5- CATTTGAATCAGGAAGCTGC-3 | 5-CCCTTCCTTCGAAATGCAAT-3 | COX2 |
| 5-CAGGGGTGGTTATTGCATCT-3 | 5-GAGACTTGCCTGGTGAAAAT-3 | IL-6 |
| 5-TTTTTTGCTGTGAGTCCCGG-3 | 5-GGACAAGCTGAGGAAGATGGC-3 | IL1-β |
| 5-GAAAGGAGAAGAGCCTGAAGTG-3 | 5- TGCCCAAGAATAGATGCTGAC-3 | MMP2 |
| 5-TTCAGGGCGAGGACCATAGAGG-3 | - CTTCTGCCCGGACCAAGGATAC-3 | MMP9 |
| 5- TGTTCCTCTATGGGGTCGTCA-3 | 5- ACTTGGCCCAGTGGGTTTT-3 | Survivin |
| 5- TGGCTTAGTGGCAGAGTCAG-3 | 5- CAGGAAATCCTCAGCAACAAAA-3 | Ki-67 |
