# Supplementary material for: Design, synthesis and biological evaluation of a new thieno[2,3-d]pyrimidine-based urea derivative with potential antitumor activity against tamoxifen sensitive and resistant breast cancer cell lines
Source: J Enzyme Inhib Med Chem. 2020 Aug 11;35(1):1641–56. doi: 10.1080/14756366.2020.1804383 (PMC7470147; doi:10.1080/14756366.2020.1804383)
Supplement: Supplemental Material [file IENZ_A_1804383_SM7703.zip › SVI.pptx]

## Slide 1
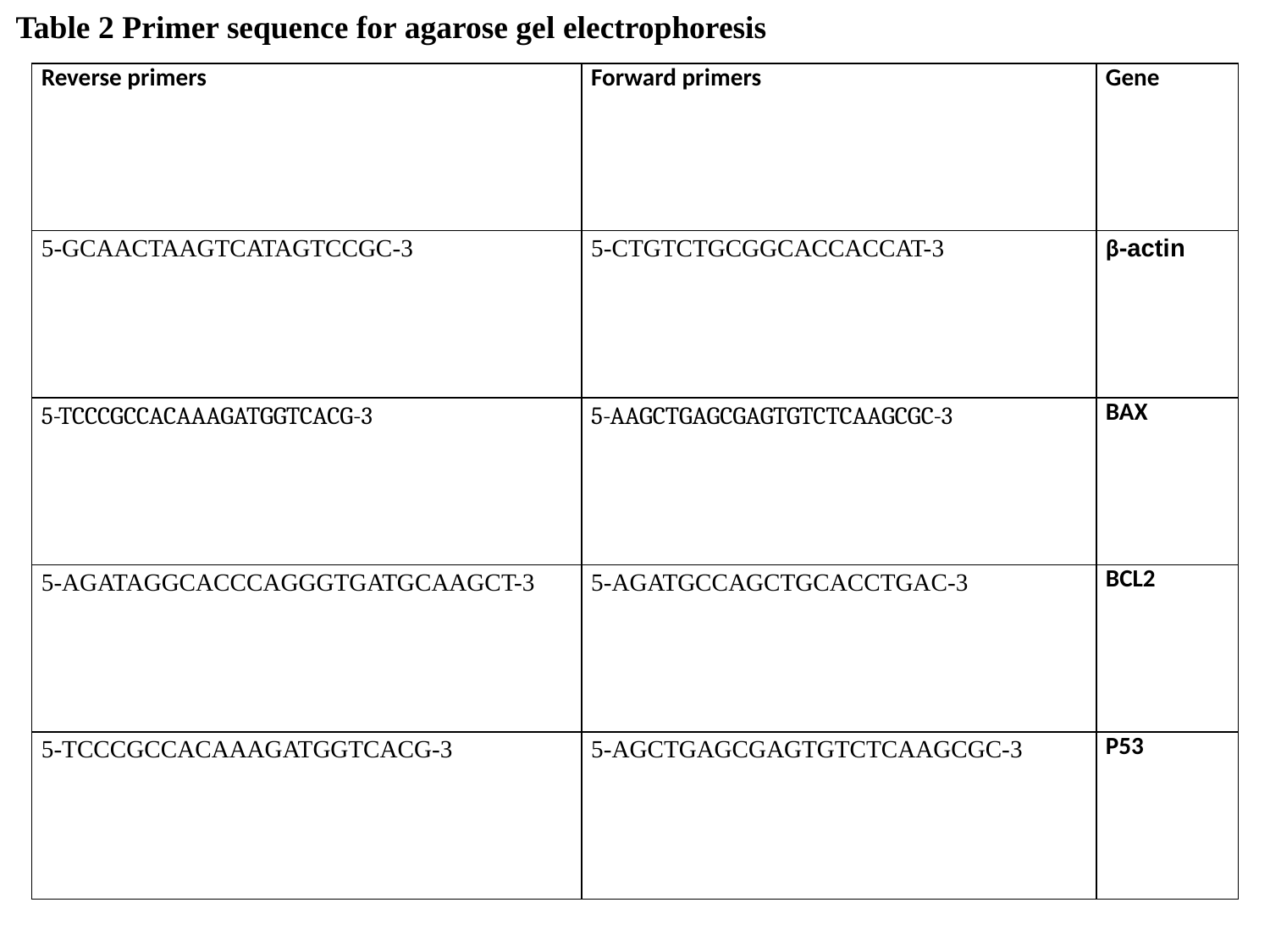

Table 2 Primer sequence for agarose gel electrophoresis
| Reverse primers | Forward primers | Gene |
| --- | --- | --- |
| 5-GCAACTAAGTCATAGTCCGC-3 | 5-CTGTCTGCGGCACCACCAT-3 | β-actin |
| 5-TCCCGCCACAAAGATGGTCACG-3 | 5-AAGCTGAGCGAGTGTCTCAAGCGC-3 | BAX |
| 5-AGATAGGCACCCAGGGTGATGCAAGCT-3 | 5-AGATGCCAGCTGCACCTGAC-3 | BCL2 |
| 5-TCCCGCCACAAAGATGGTCACG-3 | 5-AGCTGAGCGAGTGTCTCAAGCGC-3 | P53 |
