# Supplementary material for: Design, synthesis and biological evaluation of a new thieno[2,3-d]pyrimidine-based urea derivative with potential antitumor activity against tamoxifen sensitive and resistant breast cancer cell lines
Source: J Enzyme Inhib Med Chem. 2020 Aug 11;35(1):1641–56. doi: 10.1080/14756366.2020.1804383 (PMC7470147; doi:10.1080/14756366.2020.1804383)
Supplement: Supplemental Material [file IENZ_A_1804383_SM7703.zip › SVIII.pdf]

Calibration curve of VEGF

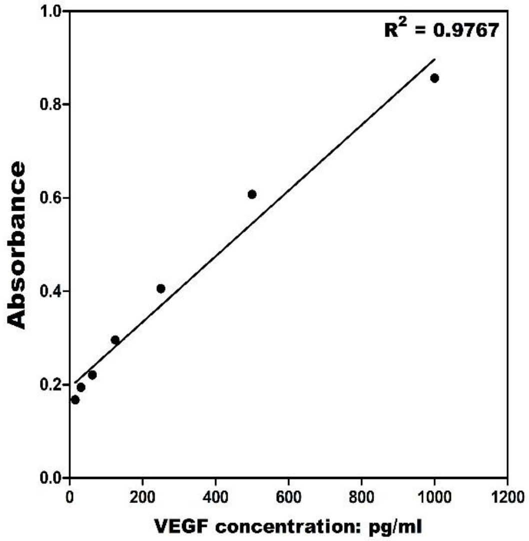

Slope	0.0007037 ± 0.00004862
Y-intercept when X=0.0	0.1933 ± 0.02122
X-intercept when Y=0.0	-274.7
1/slope	1421
95% Confidence Intervals	
Slope	0.0005787 to 0.0008287
Y-intercept when X=0.0	0.1388 to 0.2479
X-intercept when Y=0.0	-411.5 to -174.3
Goodness of Fit	
r^2	0.9767
Sy.x	0.04268
Is slope significantly non-zero?	
F	209.5
DFn, DFd	1.000, 5.000
P value	< 0.0001

Calibration curve of MDA

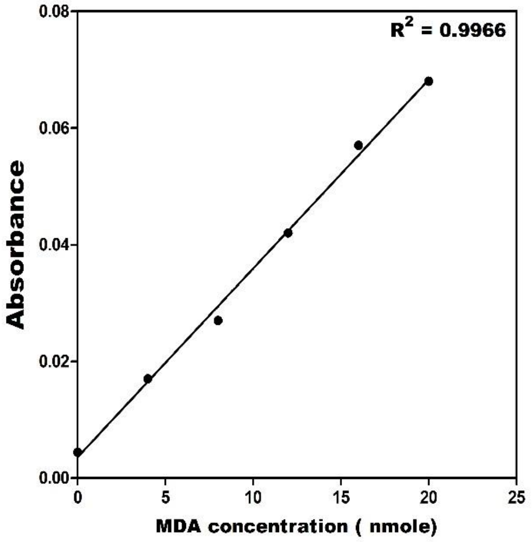

Slope	0.003236 ± 0.00009422
Y-intercept when X=0.0	0.003543 ± 0.001141
X-intercept when Y=0.0	-1.095
1/slope	309.1
95% Confidence Intervals	
Slope	0.002974 to 0.003497
Y-intercept when X=0.0	0.0003752 to 0.006710
X-intercept when Y=0.0	-2.227 to -0.1087
Goodness of Fit	
r^2	0.9966
Sy.x	0.001577
Is slope significantly non-zero?	
F	1179
DFn, DFd	1.000, 4.000
P value	< 0.0001

Calibration curve of GSH

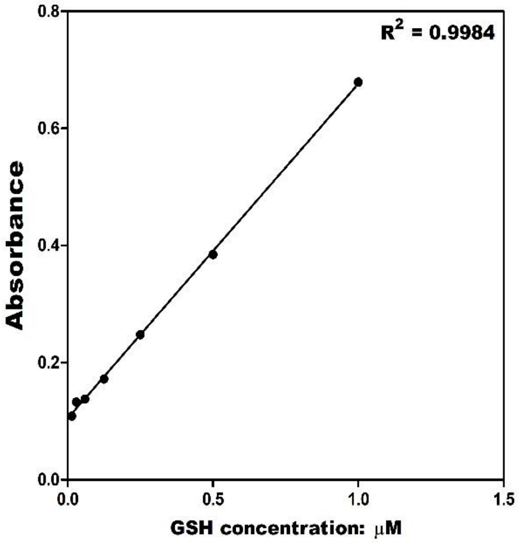

Slope	0.5715 ± 0.007167
Y-intercept when X=0.0	0.1046 ± 0.003127
X-intercept when Y=0.0	-0.1831
1/slope	1.750
95% Confidence Intervals	
Slope	0.5531 to 0.5899
Y-intercept when X=0.0	0.09659 to 0.1127
X-intercept when Y=0.0	-0.2020 to -0.1651
Goodness of Fit	
r^2	0.9992
Sy.x	0.006300
Is slope significantly non-zero?	
F	6359
DFn, DFd	1.000, 5.000
P value	< 0.0001

Calibration curve of NO

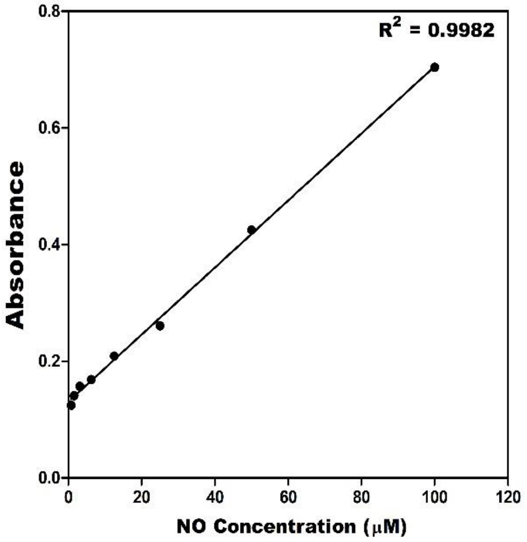

Slope	0.005744 ± 0.00009482
Y-intercept when X=0.0	0.1308 ± 0.003871
X-intercept when Y=0.0	-22.78
1/slope	174.1
95% Confidence Intervals	
Slope	0.005512 to 0.005976
Y-intercept when X=0.0	0.1214 to 0.1403
X-intercept when Y=0.0	-25.18 to -20.52
Goodness of Fit	
r^2	0.9984
Sy.x	0.008676
Is slope significantly non-zero?	
F	3670
DFn, DFd	1.000, 6.000
P value	< 0.0001

Caliberation curve of PGE2

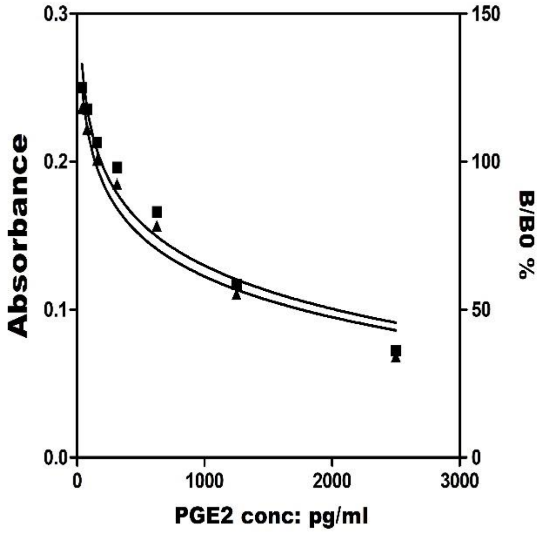

Yintercept	198.2	0.4201
Slope	-45.67	-0.09681
Std. Error		
Yintercept	11.89	0.02522
Slope	4.634	0.009827
95% Confidence Intervals		
Yintercept	167.6 to 228.8	0.3552 to 0.4849
Slope	-57.59 to -33.76	-0.1221 to -0.07155
Goodness of Fit		
Degrees of Freedom	5	5
R^2	0.9511	0.9510
Absolute Sum of Squares	272.7	0.001226
Sy.x	7.385	0.01566
