# Supplementary figures and images for: Design, synthesis and biological evaluation of a new thieno[2,3-d]pyrimidine-based urea derivative with potential antitumor activity against tamoxifen sensitive and resistant breast cancer cell lines
Source: J Enzyme Inhib Med Chem. 2020 Aug 11;35(1):1641–56. doi: 10.1080/14756366.2020.1804383 (PMC7470147; doi:10.1080/14756366.2020.1804383)

## Slide 1
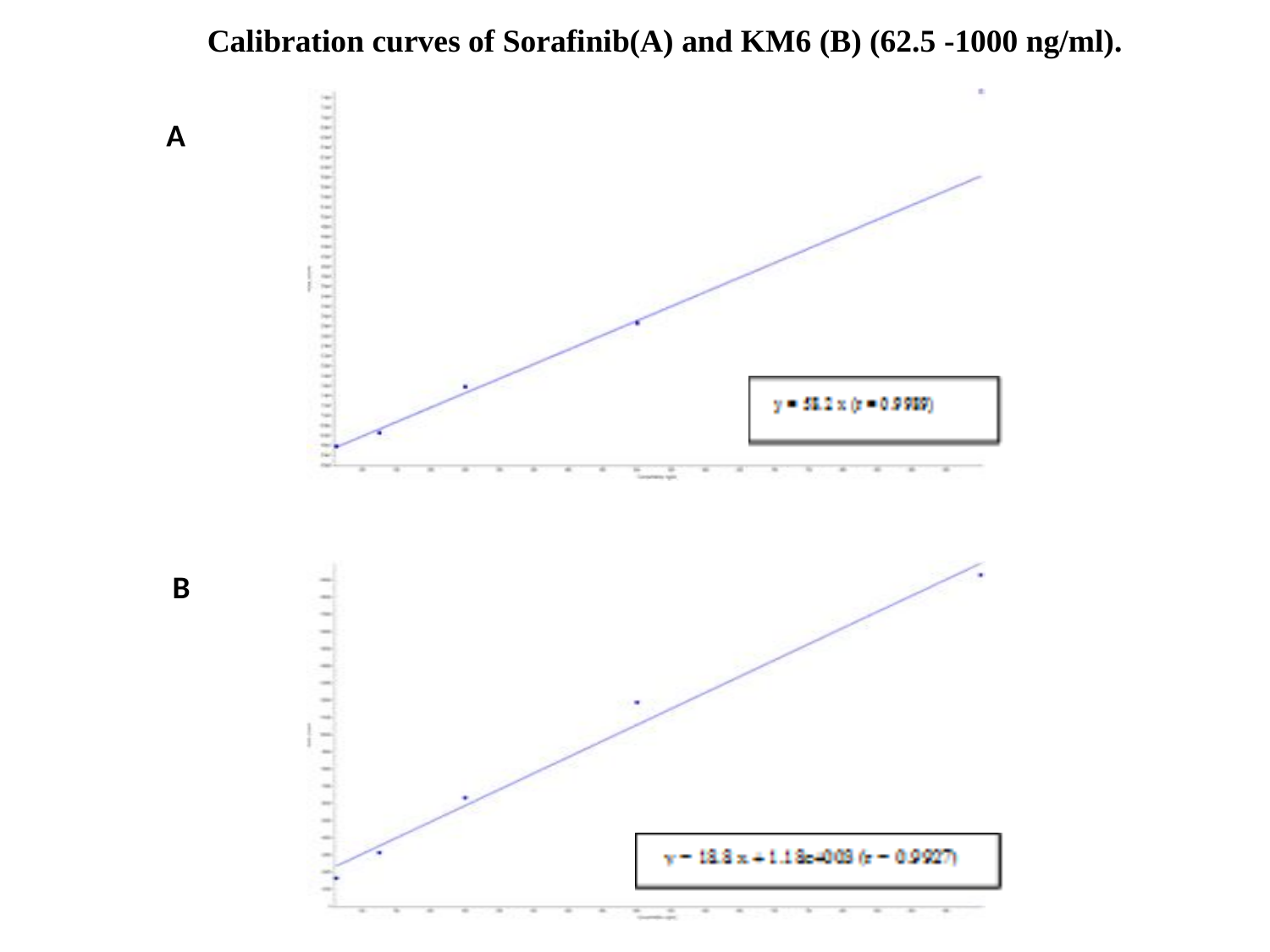

Calibration curves of Sorafinib(A) and KM6 (B) (62.5 -1000 ng/ml).
A
B

## Slide 2
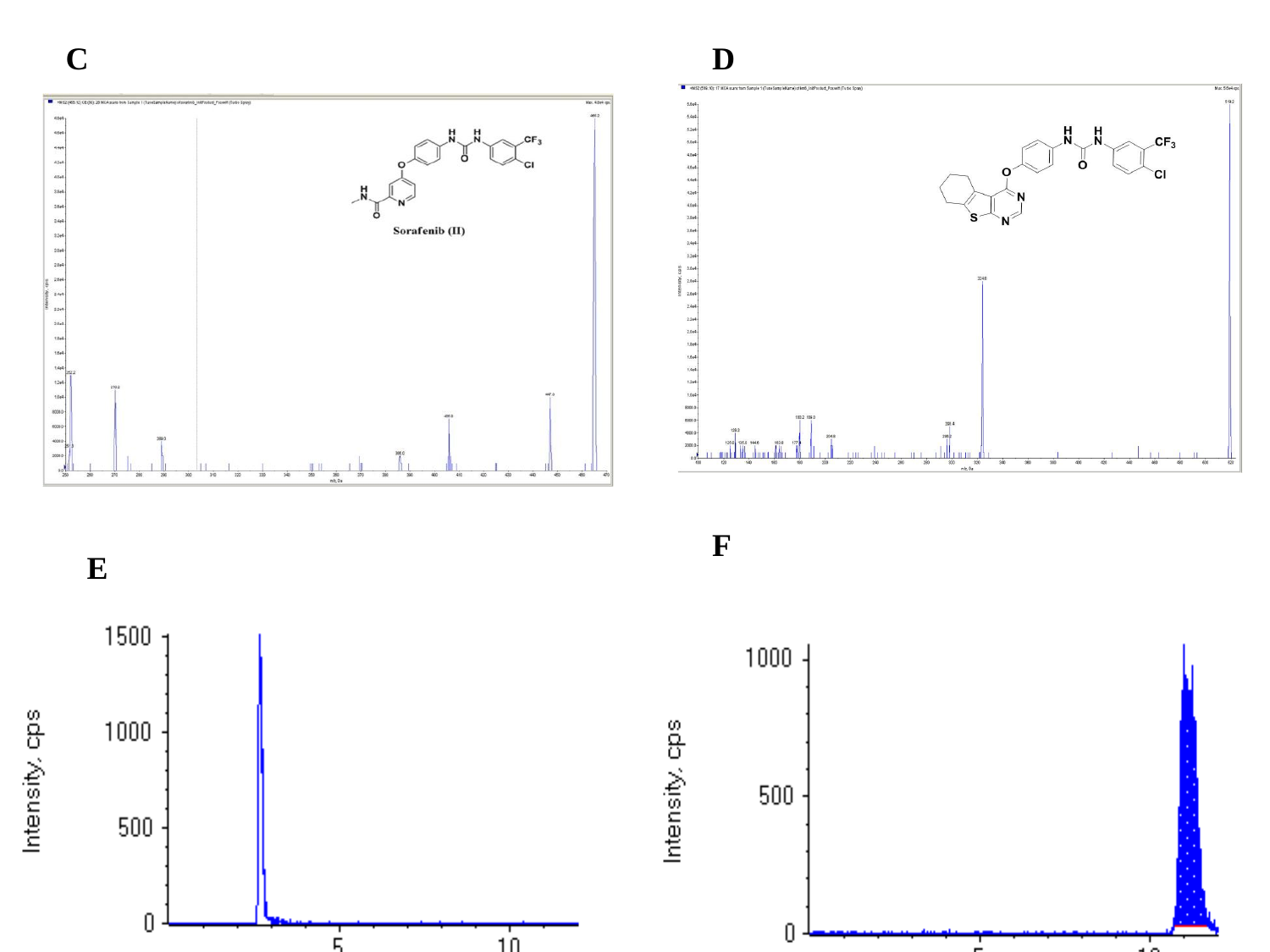

C
D
F
E

Supplement: Supplemental Material [file IENZ_A_1804383_SM7703.zip › SI.pptx]

## Slide 1
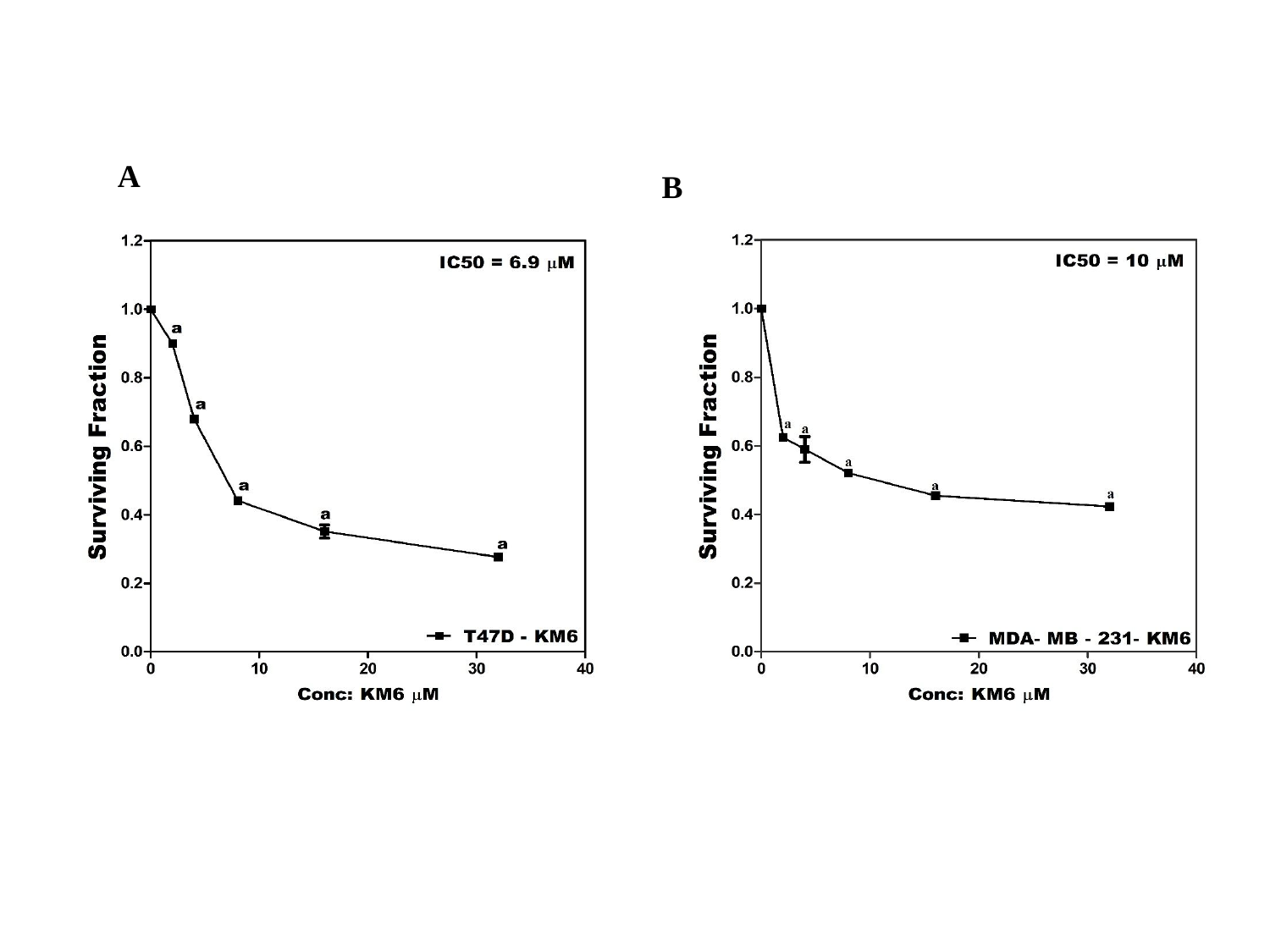

A
B

Supplement: Supplemental Material [file IENZ_A_1804383_SM7703.zip › SVII.pptx]

## Slide 1
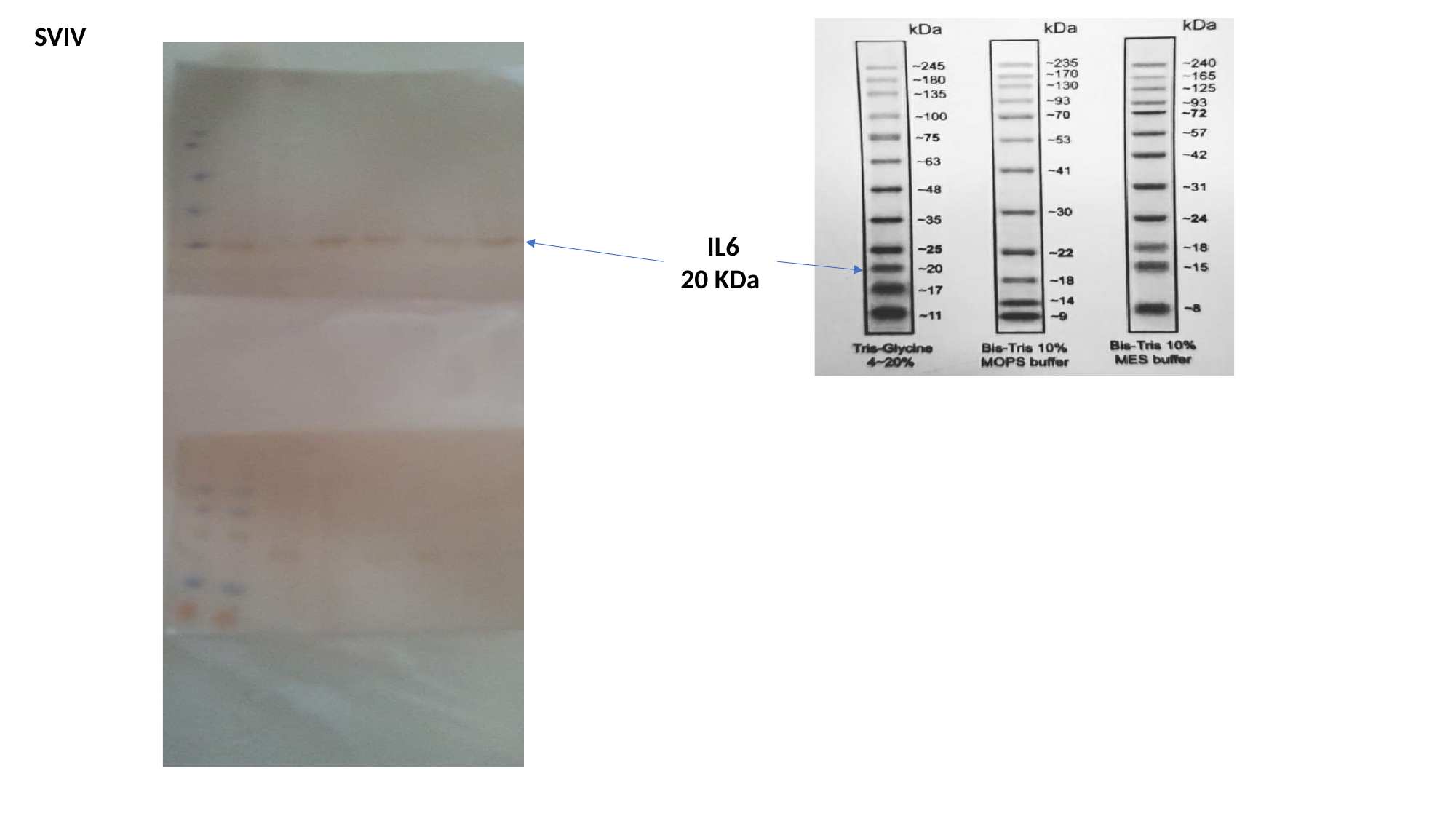

SVIV
 IL6
20 KDa

## Slide 2
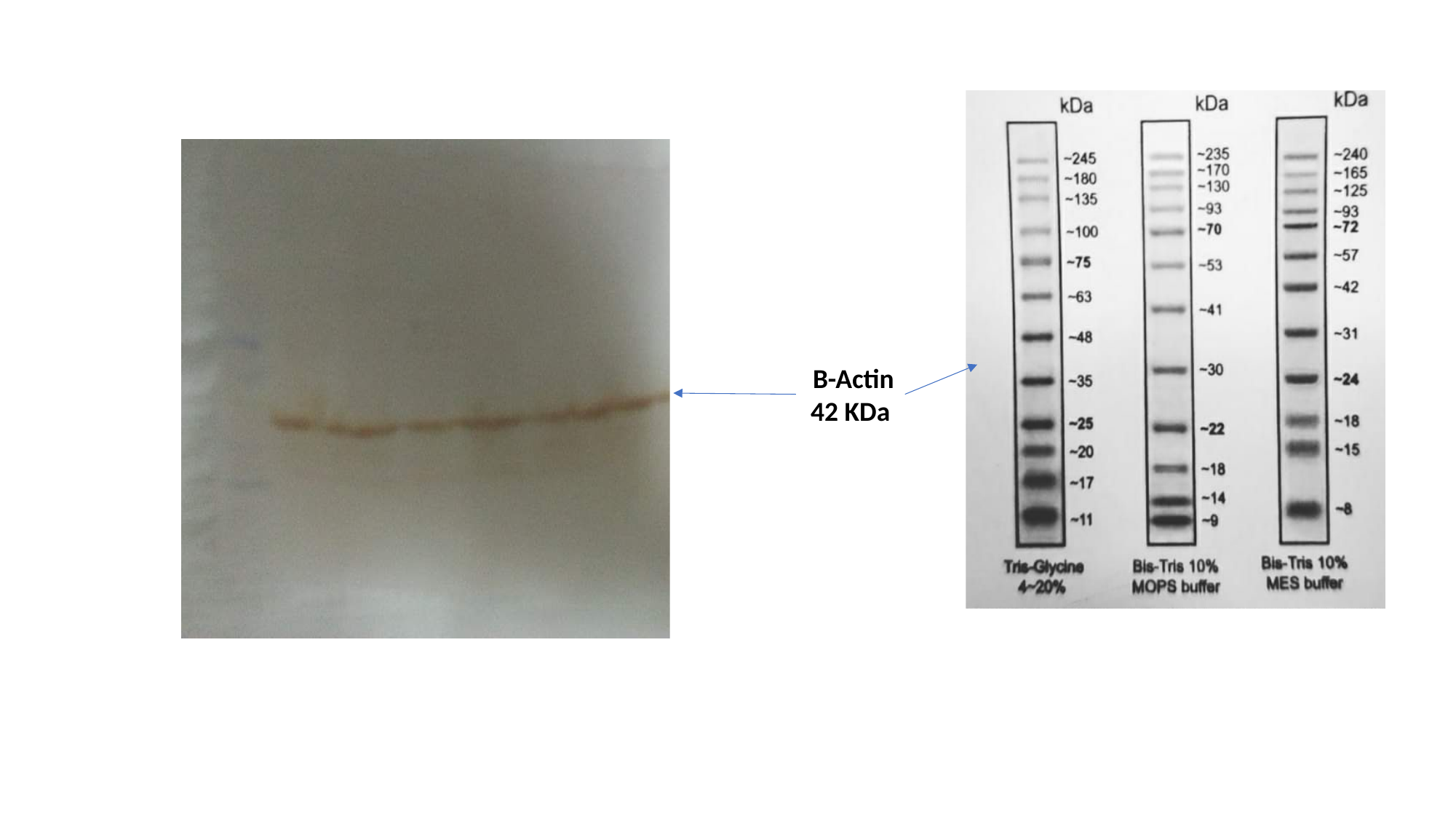

B-Actin
42 KDa

Supplement: Supplemental Material [file IENZ_A_1804383_SM7703.zip › SVIV.pptx]

## Slide 1
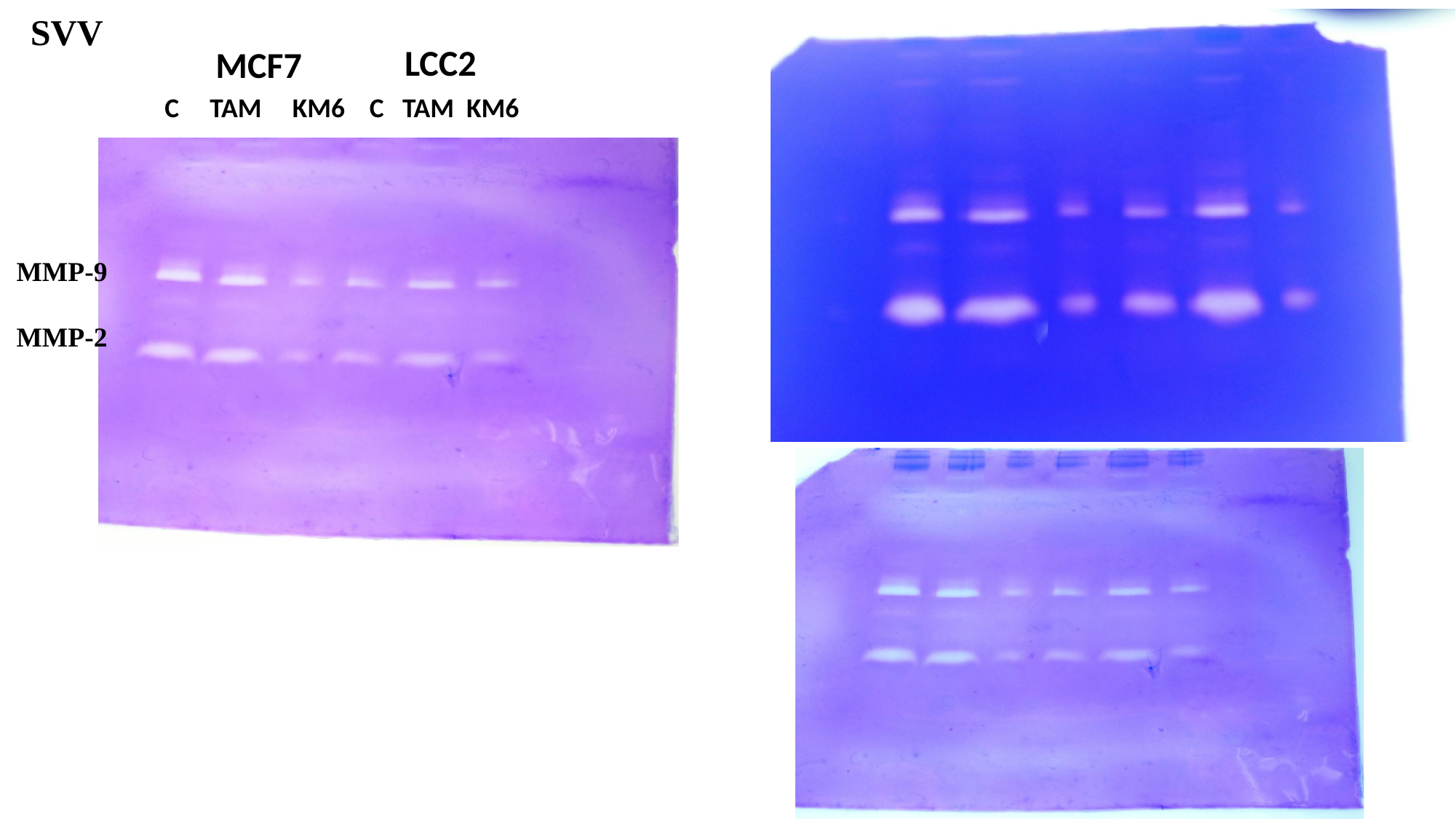

SVV
LCC2
MCF7
 C TAM KM6 C TAM KM6
MMP-9
MMP-2

Supplement: Supplemental Material [file IENZ_A_1804383_SM7703.zip › SVV.pptx]
